# Blockchain as the “trust-building machine” for supply chain management

**DOI:** 10.1007/s10479-022-04868-0

**Published:** 2022-08-05

**Authors:** Kongmanas Yavaprabhas, Mehrdokht Pournader, Stefan Seuring

**Affiliations:** 1 Department of Management and Marketing, The University of Melbourne, Level 10, The Spot, 198 Berkeley Street, Melbourne, VIC 3010 Australia; 2 Chair of Supply Chain Management, Faculty of Business and Economics, The University of Kassel, Kassel, Germany

**Keywords:** Blockchain, Trust, Supply chain, Systematic review

## Abstract

This paper aims to investigate the impact of blockchain application on trust levels in supply chains. Through the systematic review of the relevant literature, three dimensions of trust, i.e., the trustor–trustee perspective, forms of trust, and time orientation, are investigated. Our findings show that, first, there are three pairs of trustors and trustees involved in blockchain implementation: (a) the user and the blockchain, (b) two supply chain partners, and (c) the consumer/public and a supply chain unit. Second, the two forms of trust, namely cognition-based and institution-based trust, are likely to be enhanced by blockchain execution, while affect-based trust may not be directly impacted by the technology. Third, the presence of blockchain technology would facilitate swift trust-building between unknown supply chain partners under specific circumstances. Moreover, we also find contradicting assertions among scholars on the implications of blockchain for trust in supply chains. While some studies pointed out that blockchain will enable a trustless trusted scheme, others expected the reinforcement of interorganizational trust. To test these assertions, we develop the blockchain-entrusted supply chain models to present the three-step process of how trust is developed through the blockchain and diffused to supply chain partners and external stakeholders.

**Supplementary Information:**

The online version contains supplementary material available at 10.1007/s10479-022-04868-0.

## Introduction

Although trust has been considered one of the key success factors in supply chain management (e.g., Brinkhoff et al., 2015; Kwon & Suh, 2004), in recent years, global supply chains across numerous industries have experienced the issue of trust deterioration (Guenther, 2020). This is despite the fact that across industries, CEOs and executives unanimously believe maintaining and increasing trust in supply chain relationships is at the core of successful supply chain operations (Rajah, 2019). To address this concern, supply chains globally have started embracing technology-based, trust-building remedies, such as blockchain technology (Sneader & Sternfels, 2020). Due to its capability to enhance information authenticity and transparency, blockchain is believed to have the promising potential to radically transform the supply chain trade paradigm into a trusted ecosystem of exchange. Moreover, and accelerated by the recent COVID-19 disruption to supply chains, blockchain-enabled trusted supply chains have triggered interest from academics (Nguyen et al., 2020).

Academic literature has therefore initiated investigations into how to build trust through blockchain technology (e.g., Howson, 2020) and address the following question:

How can blockchain technology impact trust in supply chain management?

While one group of academic thought leaders argue that the execution of blockchain will enable firms to trade in a trustless ecosystem where there is no need for building trusted relationships between trading partners (e.g., Asante et al., 2021; Kumar et al., 2020), others believe that blockchain will enhance trust between supply chain partners and create a trusted ecosystem of exchange (e.g., Centobelli et al., 2021; Di Vaio & Varriale, 2020). Caldarelli et al. (2020) explicitly stated that early blockchain literature believed that blockchain is a means for creating trust, but more recently, scholars seem to focus on blockchain capabilities to enable transactions performed in a trustless environment. Likewise, while some academics show that blockchain installation can generate trust which will subsequently add to a trusted relationship between trading parties (Joo & Han, 2021), others believe that trust generated from the blockchain cannot directly transfer into trading partners, and trust management between parties is still required (Kopyto et al., 2020). Whether these seemingly contrasting views can converge to a single trust-based framework for application of blockchains to supply chains is what we are aiming to address in this paper.

To date, there has not been a comprehensive review that synthesizes the state-of-the-art body of knowledge regarding blockchain implications on trust for supply chain management. The current study aims at filling this gap by conducting a systematic literature review of blockchain and trust in supply chains. We initially use the three dimensions of trust from management research, namely the trustor–trustee perspective, a form of trust, and temporal orientation, as a framework for content analysis. We adopt a combination of inductive and deductive approaches to analyse content and gain insights from our selection of 94 relevant academic publications published between 2018 and 2021. Subsequently, we developed a three-step trusted ecosystem model of the blockchain-based supply chain that demonstrates how trust is formed by adopting blockchain to supply chains and disseminating to other supply chain stakeholders. The proposed models are developed in three variations to consolidate and converge the aforementioned contradicting views of blockchain application to supply chains as well as making a case for swift trust formation under certain circumstances in supply chains.

The remainder of the paper is organized as follows: first, we provide an overview of the literature on the definitions and assessment of trust in supply chains as well as a theoretical background of three dimensions of trust leading to Sect. 3, where we outline the underlying methodology. In Sect. 4 we discuss the outcome of the literature review regarding the blockchain implication on trust in supply chains and subsequently develop three models of the blockchain-entrusted supply chain. Our contributions to theory and practice are outlined in Sect. 5. Finally, we provide the conclusion of this study and several avenues for future research in Sect. 6.

## Conceptual foundation

Blockchain is a decentralized network system comprising a number of blocks, each of which is capable of storing and sharing real-time, encrypted digital information to other blocks within the network (Nakamoto, 2008). By perceiving each supply chain member as a block, hypothetically, every participant is allowed to know “who is performing what actions in which location and at what time” in real-time (Crosby et al., 2016). With this exceptional characteristic, blockchain equipment is projected to bring the superiority of system security and information visibility to supply chain operations (Carson et al., 2018). During the past three years, numerous comprehensive reviews of blockchain technology and its application to supply chains have been conducted (e.g., Chang & Chen, 2020; Luo & Choi, 2021; Müßigmann et al., 2020; Pournader et al., 2020b; Saberi et al., 2019; Wang et al., 2019a). While we refer the readers to these reviews for an in-depth discussion of blockchain application to supply chains, in this section we primarily focus on the literature on trust and trust dimensions, to be adopted as a core framework in the following sections.

### Trust in supply chain management

Trust has been considered at the core of relationship management between supply chain partners (e.g., Brinkhoff et al., 2015; Kwon & Suh, 2004). As the relationship between supply chain partners is often fragile and involves a certain level of risk in terms of opportunistic behaviour, there is a need for trust to bind these relationships and manage those risks (Spekman, 1988). When supply chain members possess a high level of trust in one another, they are likely to feature positive attributes including greater collaboration (Ha et al., 2011), operational efficiency (Ha et al., 2011), responsiveness (Handfield & Bechtel, 2002), innovativeness (Fawcett et al., 2012), flexibility and agility (Kabra & Ramesh, 2016), and resilience (Jain et al., 2017).

Trust is defined in the extant literature as “a willingness to be vulnerable” (Mayer et al., 1995), “the extent to which a person is confident in, and willing to act on the basis of, the words, actions, and decisions of another” (McAllister, 1995), “the willingness to be vulnerable under conditions of risk and interdependence” (Rousseau et al., 1998), and “confident positive expectations regarding another’s conduct” (Lewicki et al., 1998). It is noticeable that from multiple proposed definitions, trust always involves at least two actors, *a trustor* who is in a vulnerable position through risks and interdependence, and *a trustee* who is entrusted and can take advantage of the trustor by not fulfilling his or her expectation. Trustor and trustee entities can vary as trust can be bestowed upon an individual (McAllister, 1995), an organization (Kramer, 1999; Zaheer et al., 1998), political institutions (Kim, 2005), and other objects. Extending the latter definitions of trust to supply chains, the willingness to be vulnerable occurs when one entity (trustor) agrees to have confidence in the behaviours or actions of another entity (trustee) and has an expectation that the trustee will fulfill their obligations independent of the trustor’s ability to monitor or control such behaviours.

### Dimensions of trust

Existing literature indicates that trust primarily consists of three dimensions, namely *the pairs of trustor–trustees* (Janowicz & Noorderhaven, 2006), *forms of trust* (Laeequddin et al., 2010), and *time orientation* (Dubey et al., 2019). Collectively, these dimensions should further clarify “who” the trustor and trustee roles are in supply chains, as well as the forms of trust that are bestowed upon them.

- *(1) Trustor–trustee perspective.* Trust involves at least one trustor and one trustee entities, such as a buyer and a supplier. It is crucial to determine who the trustor and trustees are before conducting a trust investigation. Janowicz and Noorderhaven (2006) suggested that a change in a pair of trustors and trustees in supply chains can lead to the need for variation in the approaches for trust assessment. For instance, the conceptualization and assessment of interorganizational trust between supply chain partners (e.g., Capaldo & Giannoccaro, 2015; Janowicz & Noorderhaven, 2006) varies from the trust external consumers place on supply chain management and members (e.g., Hoejmose et al., 2012; Macready et al., 2020).
- *(2) Form of trust.* Trust in supply chain management has variations in its form (Laeequddin et al., 2010). Although forms of trust are represented in different names in previous studies in the management arena, they seem to be grounded on the three rudimentary categories namely *institution-based trust*, *cognition-based trust*, and *affect-based trust* (Lewis & Weigert, 1985; Rousseau et al., 1998; Wong et al., 2008). These three fundamental forms of trust in management research can be adopted for discussing the trust element in supply chains.
  *Institution-based trust* or *system-based trust* refers to the trust that emerges from formalized arrangements and control mechanisms which normally manifest in the form of legal systems, regulations, bureaucratic sanctions, and contracts and agreements between two parties (Lewis & Weigert, 1985; Wong et al., 2008). As institutional trust can help deter a party’s opportunistic behaviour due to law and reputational sanctions, a trustor is likely to bestow a higher level of trust on a trustee in the presence of such arrangements and control mechanisms (Rousseau et al., 1998).
  *Cognition-based trust* or *rational trust* refers to trust that involves high rationality and low emotionality through which a trustee shows “good reasons” and evidence to justify their intentions to perform an action which the trustor perceives as beneficial (Lewis & Weigert, 1985; Wong et al., 2008). Basically, a trustor bestows this type of trust based on their calculation of the costs and benefits as well as their prediction of the outcomes and the possibility that a trustee will complete their obligations as agreed (Doney & Cannon, 1997). Therefore, credible information from trustees regarding their competencies and intentions is paramount in the formation of cognition-based trust (Rousseau et al., 1998; Wong et al., 2008).
  *Affect-based trust* or *emotional trust* is signified by high emotionality and low rationality. It refers to the trust that involves affectional bonds and attachment between the trustor and trustee (Lewis & Weigert, 1985; Rousseau et al., 1998). McAllister (1995) also indicated that a trustor may manifest this type of trust by showing intention to provide extra help and assistance to a trustee without gaining anything in return. Affect-based trust is typically considered as traditional trust between partners within supply chains (Wong et al., 2008).
- *(3) Temporal orientation trust.* In management research, there is a specific type of trust that can be built rapidly in certain conditions, in contrast to traditional trust that develops over the long term (Meyerson et al., 1996; Robert et al., 2009). Long-term versus swift trust-building practices are included within the domain of temporal orientation. Originally coined by Meyerson et al. (1996), *swift trust* is a specific type of trust that can be established rapidly for a temporary group of people or entities to accomplish task-specific objectives in a short period of time. It normally stems from the combination of current circumstances and conditions evaluated by the trustor including group members’ reputations, assigned responsibilities, consistent information exchange, and committed norms, and rules. Swift trust has recently gained the increasing attention of supply chain scholars interested in increasing supply chain coordination and agility (e.g., ; Dubey et al., 2019). At the other end of the spectrum, *long-term oriented trust* is the conventional concept of trust that is built over a certain period of time. Long-term developmental trust typically requires a combination of different forms of trust (Rousseau et al., 1998). A summary of three dimensions of trust and the categories in each dimension adopted from management research are provided in Table 1.

**Table 1:** Summary of trust dimensions, descriptions, and categorizations

Trust dimensions	Description	Categories
Trustor–trustee perspective	Identify two actors of trustor and trustee	
Form of trust	Identify different forms of trust bestowed from trustor to trustee	Institution-based trust Cognition-based trust Affect-based trust
Temporal orientation	Identify the required time and situation in which trust can be developed	Swift trust Long-term oriented trust

## Methodology

In order to investigate the literature surrounding the impact of blockchain on trust in supply chains, we opted for a systematic literature review approach. According to Tranfield et al. (2003), there are two main objectives for conducting a literature review: (1) drawing connections from fragmented knowledge pieces and consolidating the intellectual frontier of a certain research area, and (2) identifying knowledge gaps and uncharted territories waiting for future investigation. Prior studies in the supply chain management field have used systematic literature review to serve two such purposes in a variety of research arenas such as risk management in supply chains (Ho et al., 2015; Pournader et al., 2020a), sustainability issues in supply chain management (Yawar & Seuring, 2017) and blockchain in supply chains (Pournader et al., 2020b; Saberi et al., 2019).

In this regard, we followed the four steps of a systematic literature review process suggested by Bryman (2016): *source identification, source selection*, *source evaluation,* and *content analysis*.

### Source identification and selection

We opted to use the Scopus search engine (www.scopus.com) which has been used for previous systematic literature reviews in supply chain management arenas (e.g., Pournader et al., 2020a; Fahimnia et al., 2019; Pournader et al., 2021; Wang et al., 2019a). Based on previous studies and our personal experience, Scopus has provided exhaustive coverage of papers published in the fields of business, economics, management, and social sciences compared to other search engines such as Google Scholar and Web of Science (Martín-Martín et al., 2018; Mongeon & Paul-Hus, 2016; Pournader et al., 2020b). We conducted our search using the following three systematic steps:

Step 1: We added selective keywords to the following search algorithm to look for keywords in titles, abstracts and keywords of the sources:

TITLE-ABS-KEY (“blockchain” OR “distributed ledger” OR “distributed technology” AND “supply chain” OR “supply network” OR “value chain” OR “logistic” AND “trust*”).

To ensure maximum coverage of the literature on blockchain and trust in supply chains, the search strings are adopted from previous systematic literature reviews of blockchain for supply chains (Pournader et al., 2020b; Wang et al., 2019a) and those of trust in supply chains (Delbufalo, 2012; Paluri & Mishal, 2020) that are published in quarter one (Q1) of the 2020 SCImago Journal Rank (SJR). We combined the search strings relate to blockchain and supply chains from the systematic literature reviews of blockchain for supply chains with the keywords of trust from the systematic literature reviews of trust in supply chains. The search continued until the end of 2021. There were 699 records shown in the document records.

Step 2: We then limited our search to articles and articles in the review which were published in English. The search results presented 289 records.

Step 3: We assessed the titles, abstracts, and keywords of each article to determine its suitability for inclusion. If required, we also closely investigated other sections of the articles including discussions, findings, and conclusions. Articles that were aligned with our two selection criteria are selected. First, the chosen articles needed to be related to the blockchain implication of trust in supply chains. Articles that were selected need to clearly state supply chain activities that are supported by blockchain or the purposes of blockchain implementation that need to be related to supply chain management (Bryman, 2016). Articles that used blockchain for other purposes such as using the technology to explain cryptocurrencies in other contexts beyond supply chain management were excluded. It is worth noting that we only included articles that investigated “the impact of blockchain adoption on trust” and excluded articles that examined “the impact of trust on blockchain adoption” as our objective of this study focuses on the implication or potential impact of blockchain on trust in supply chains after being adopted.

Second, the selected articles needed to be published in Q1 of the 2020 SJR such as *International Journal of Production Research, Supply Chain Management: An International Journal, International Journal of Information Management, Decision Sciences, and IEEE Access*. The SJR indicator is a widely accepted measurement of journal rankings which are evaluated based on both the journal’s number of citations as well as the journal’s prestige (Mañana-Rodríguez, 2015). After applying this screening method, we eventually came up with 94 sample articles.

Thus, using this method we further minimized the selection bias in the source identification process by enabling a recursive and iterative process of reviewing and refining search keywords (Saunders et al., 2009). Furthermore, each of the coauthors independently reviewed the articles and double-checked their screening outcomes and came to a consensus, further decreasing the selection bias.

### Source evaluation

The outcome of the bibliometric analysis showed that all 94 articles selected were published in 2018 (two articles), 2019 (11 articles), 2020 (32 articles), and 2021 (49 articles). *IEEE Access* with 20 articles contained a high number of publications, followed by *the International Journal of Production Research* and *Sustainability* with six articles. The remaining journals that have more than two publications are *Computers & Industrial Engineering* with five articles, *Journal of Cleaner Production* with three articles, and *International Journal of Information Management* with three articles published. It can be said that the published journals are quite scattered as most papers came from different journals. This is possibly due to the investigation of blockchain implications of trust in different supply chains.

### Content analysis

We adopted a combination of inductive and deductive approaches to conduct content analysis on the selected pool of articles. Initially, we utilized the theoretical ground of three trust dimensions as our underlying framework for analyzing the body of literature.

#### Inductive approach for the trustor–trustee perspective

As Janowicz and Noorderhaven (2006) suggested that there are differences in the trust element between different pairs of trustors and trustees, yet no specific pairs of relationships are provided, there is a multiplicity of possibilities for pairs of trustors and trustees. As the trustor–trustee perspective does not have any predefined category, we utilized the inductive approach to extract new themes and subthemes from selected publications and to develop new main categories (Seuring & Gold, 2012; Tranfield et al., 2003). We followed the two-step approach recommended by Seuring and Gold (2012) to establish the basic framework of categories that emerged from the literature analysis and to inductively refine each category during the coding process. We adopted the three-step coding process suggested by Yin (2015), starting with open coding to extract important quotes from literature, followed by axial coding and selective coding to form subthemes and themes within the trustor–trustee framework, respectively. We performed the analysis back and forth between literature and our emergent themes to reach data saturation. With this rigorous inductive approach, the literature review can be deemed as an effective approach in new theory development (Seuring et al., 2020).

#### Deductive approach for the forms of trust and time orientation

For both dimensions of the forms of trust and time orientation, as predetermined categories are from management literature, we employed a deductive approach to link those existing categories to a body of literature reviewed in the blockchain context. This approach allowed us to simply borrow those categories as referencing codes in the coding process. Specifically, we used the categories of *institution-based trust*, *cognition-based trust* and *affect-based trust* to code forms of trust found in selected articles. The form of trust was either typically latent or not manifested clearly in the literature. In other words, we needed to delicately interpret the meanings contained within the articles to extract the exact forms of trust that each publication referred to. For the time orientation dimension, we used the categories of *swift trust* and *long-term oriented trust* in the coding process. This deductive approach to borrow theory outside the field of supply chain management to enrich phenomena in supply chains is considered a justified approach for theory extensions (Seuring et al., 2020). The summary of the coding process in our literature review is outlined in Table 2.

**Table 2:** Summary of the content analysis and coding process

Approach	Trust dimensions	Predefined categories	Coding process
Inductive	Trustor–trustee perspective		Open coding—to extract important quotes, Axial coding—to form categories as subthemes, Selective coding—to group subthemes into the main themes as new categories
Deductive	Forms of trust	Institution-based trust Cognition-based trust Affect-based trust	Use predefined categories to code selected publications
	Time orientation	Swift trust Long-term oriented trust	Use predefined categories to code selected publications

### Material evaluation

To ensure the reliability and validity of data analysis, we adopted the following measures. First, we included all the coauthors in the process of content analysis. Specifically, during the coding process, each coauthor performed the coding independently. Using the judgement of multiple coauthors to interpret latent content in a body of literature, together with intense discussions among the research team, broadly enhanced the internal validity and reliability of the findings (Duriau et al., 2007). Second, we adopted a theoretical categorization scheme with clear definitions from management scholarship for both inductive and deductive approaches, and also utilized the predefined categories for deductive analysis. The analysis based on these theoretical grounds increased the reliability of the coding (Seuring & Gold, 2012). In addition, the de-contextualisation and focus on theoretically-based trust in our coding process further strengthened the generalisability and thus external validity (Avenier, 2010). Finally, we followed *the discursive alignment of interpretation* approach suggested by Seuring and Gold (2012), in which there are inconsistent judgements occurring during the coding process. This approach allowed us to resolve contentious issues by allowing the research team to gradually and deliberately reinterpret the meaning embodied in the texts which referred to latent contents, until consensus was reached. After completing this iterative process of content analysis, we summarized the explanation of blockchain and trust in supply chains from all 94 selected articles. This is detailed in online Appendix A.

## Blockchain and trust in supply chains

From the inductive analysis, we extracted three emergent pairs of relationships in the blockchain and supply chain literature with respect to the trustor–trustee perspective. We also found two emerging subthemes under one pair of relationships. As for deductive analysis, we adopted the proposed trust categories in Sect. 2 to explain the impact of blockchain on trust in supply chains across the dimensions of the form of trust and time orientation.

### The trustor–trustee perspective

Content analysis revealed three distinct themes as the pairs of relationships in blockchain-enabled trust in supply chains contexts, i.e., a user and the blockchain, two supply chain partners, and consumers/public and the supply chain entity. Moreover, for the pair of supply chain partners, we found two subthemes that showed the main disparities in implications of blockchain adoption and the actors who assume the roles as trustors and trustees.

In terms of the number of papers, 78 papers investigated the impact of blockchain on two supply chain partners, in which 41 articles revolved around the first subtheme of *Trustless trusted scheme (TTS),* in which two supply chain partners are trustors and the blockchain solution acts as a trustee. The other 38 articles discussed the impact of blockchain within the second subtheme of *Interorganizational trust reinforcement (ITR)*, in which interorganizational trust between supply chain partners are enhanced and one supply chain partner acts as a trustor while the other partner acts as a trustee. In addition, 57 papers mentioned the direct impact of blockchain on users’ trust and 31 articles examined the impact of the blockchain supply chain on consumers and the public. The emergent three pairs of trustor–trustees showed different levels of units of analysis. Specifically, the blockchain solution and its user pairs, as well as the two supply chain partners pairs demonstrated the impact of blockchain on an organizational level, while the consumers/public and a unit of supply chain pairs demonstrated the impact of blockchain adoption on supply chain level. The summary of emergent themes under the framework of the trustor–trustee is shown in Table 3.

**Table 3:** Summary of emergent themes within the framework of trustor–trustee perspective

Emergent themes	Subthemes	Trustor	Trustee	No. of papers	Unit of analysis
The pair of users and blockchain		The system users	Blockchain	57	Organization
The pair of two supply chain partners	Trustless trusted scheme (TTS)	Two supply chain partners	Blockchain	41	Organization
	Interorganizational trust reinforcement (ITR)	A supply chain partner	A supply chain partner	38	
The pair of consumers/public and a unit of supply chain		Consumers and the public	A unit of supply chain	31	Supply chain

#### User and blockchain pairs

The trustor–trustee pair belongs to the trustor of an organization that utilizes the blockchain and the trustee of such a system. The reviews of existing literature suggest that from the users’ viewpoint, the capability of the blockchain platform to fulfill its obligation of storing and exchanging information is enhanced compared to the traditional interorganizational systems (Garrard & Fielke, 2020). For instance, Hu et al. (2021) suggested that with blockchain, the trust perceived by users is enhanced in both the perspective of system trust (trust in labelling and certification processes, normally rooted in institutions) and technology trust (the technological system’s performance and quality attributes). We coined this as *the trust of users in the blockchain-based system*. The enhanced trust stems from three main sources of the blockchain’s reinforced capacity, namely the superiority of information security (e.g., Kayikci et al., 2020; Treiblmaier & Sillaber, 2021), faster data transmission speed (e.g., Palas & Bunduchi, 2020; Tseng & Shang, 2021), self-executing governance, and monitoring (e.g., Qian & Papadonikolaki, 2020; Velmovitsky et al., 2021).

##### Superiority of information security

As blockchain ledgers are encrypted and only allow authorized parties to access records, they cannot be tampered with if all relevant parties do not agree (Ahmad et al., 2021). Using blockchains, attempts to corrupt or falsify data within the system will be rendered unsuccessful (Köhler & Pizzol, 2020). Furthermore, as information in the system is copied to every digital ledger in the blockchain network, hacking or manipulating all ledgers is almost impossible (Babich & Hilary, 2020; Musamih et al., 2021). This superiority of the blockchain security mechanism is expected to substantially reduce fraudulent activities that might occur in an interorganizational setting, i.e., the supply chain (Cha et al., 2020; Garrard & Fielke, 2020). Therefore, with the application of blockchain, users are likely to show greater trust in system security and data within the system (Viriyasitavat et al., 2019).

#### Faster data processing and transmission

As blockchain operation is governed by self-executing smart contracts, the communication process between different entities in the blockchain is subsequently automated and streamlined (Wang et al., 2019b). Theoretically, data is communicated on a nearly real-time basis within the blockchain (Crosby et al., 2016). Thus, with the increase in speed and efficiency of communication and transaction processes, users are likely to view the higher capability in fulfilling transactions favourably compared to traditional systems and bestow a higher level of trust in blockchain (Palas & Bunduchi, 2020).

##### Self-executing governance and monitoring

With the application of self-executing smart contracts, blockchain users can be assured that financial transactions are automatically governed and monitored to be completed as agreed in advance between supply chain partners (Qian & Papadonikolaki, 2020). If the system is interrupted or damaged, appropriate actions will be automatically executed to rectify the issue (e.g., Alkhoori et al., 2021). With such highly reliable processing and monitoring mechanisms, it is likely that users will bestow higher trust to blockchain platforms compared to the traditional supply chain systems without smart contracts (e.g., Pawar et al., 2021).

#### Two supply chain partners pairs

The second trustor–trustee pair is the two supply chain partners. There exist discrepancies in the literature with respect to the implications of blockchain on trust in supply chain partners as well as the actors who assume the roles of trustor and trustee. Specifically, there are two distinct views, as shown in Table 4, one arguing that blockchain deployment will enable trustless trusted supply chain operation (i.e., TTS) where there is no need for interorganizational trust development, and the other school of thought arguing that blockchain adoption will initiate and reinforce trust between supply chain partners (i.e., ITR).

**Table 4:** The two distinct views of blockchain implications for trust between two supply chain partners

Distinct views	Blockchain application implications	Trustor	Trustee
Trustless trusted scheme (TTS)	There is no need for interorganizational trust development The two supply chain parties only trust blockchain Trust is redistributed and more centralized toward the operating system	Two supply chain partners	Blockchain
Interorganizational trust reinforcement (ITR)	Blockchain help initiate and reinforce interorganizational trust between two supply chain partners	A supply chain partner	A supply chain partner

*(a) Trustless trusted scheme (TTS).* Academics believe that the integration of blockchain into supply chain operating systems will allow supply chain partners to perform transactions in a trustless fashion (e.g., Treiblmaier & Sillaber, 2021; Viriyasitavat et al., 2019). Academics further elucidate that blockchain employment will eliminate the necessity of trust-building between different parties and transfer trust provided from each party toward the blockchain (Kumar et al., 2020; Pournader et al., 2020b). In other words, blockchain itself acts as the trusted third party that facilitates transactions between supply chain partners (Cha et al., 2020). In this regard, the blockchain solution is viewed as more trustworthy due to its three sources of capacity: the superiority of information security, faster data transmission speed, and self-executing governance and monitoring. Therefore, in TTS, scholars believe that with the presence of blockchain, supply chain parties do not need to trust each other as they can merely trust information and the authenticity of entries added to the system (Kumar et al., 2020; Tezel et al., 2021).

*(b) Interorganizational trust reinforcement (ITR).* In contrast to TTS, scholars argue that one of the major contributions of blockchain adoption is the enhancement of trust between different trading parties (e.g., Dubey et al., 2020b; Joo & Han, 2021). This enhanced trust is likely to be interorganizational trust—the collaborative trust between supply chain partners (Fawcett et al., 2012). The main arguments surrounding such interorganizational trust are rooted in the three reasons below.

##### Transfer of the strengthened trust in a blockchain

The leveraged trust between organizations is directly transferred from the enhanced trust in the blockchain. Since a trustee organization executes the trusted blockchain with the improved capability of fulfilling obligations, and fewer chances of hacking or fraud, a trustor party may bestow a higher level of trust to such a system operator (Surjandari et al., 2021). For instance, Hunt et al. (2021) and Shahid et al. (2020) demonstrated the utilization of a blockchain-based reputation system or a peer-rating system that trace the interaction of participants within the supply chain network and actively compute reputation scores for each of them. The calculated score then reflects an organization’s capability to complete the desired obligation. Due to the security and reliability of the blockchain-based reputation system, organizations with a higher score are perceived as more trustworthy in the eyes of their partner organizations in the supply chain. In other words, trust translates from the trustworthiness of the blockchain to trust in trading partners within the supply chain. In contrast to this view, Kopyto et al. (2020) states that trust-associated advantages of blockchain technology that are shifted into an interorganizational setting, such as a supply chain, cannot be directly transferred to the trustworthiness in the trading partner as the authenticity of data entered into the blockchain cannot be guaranteed. Blockchain can only create the cryptographic proof of digital data in the system, not the physical information which means that trust management to reduce data manipulation before entering into the system is still a prerequisite, even when a blockchain solution is adopted.

##### Information transparency

The second underlying reason for increase in interorganizational trust stems from the establishment of the information transparency paradigm and the repeated pairwise exchange of information between the two partners (e.g., Ghode et al., 2020; Li et al., 2020). Considering the two parties within the supply chain, when a trustee entity discloses and inputs the required information into the blockchain, another party as a trustor will gain more knowledge regarding the trustee’s capability to satisfy the two parties’ obligations. Therefore, they are likely to provide a higher level of trust to the trustee with more transparent information (Akkermans et al., 2004; Wan et al., 2020). As blockchain requires all participants within the network to share essential information in a real-time manner (Crosby et al., 2016), from the pairwise perspective of trading partners, the presence of blockchain then helps promoting trust bestowed from one to the other (Pan et al., 2020). For instance, Di Vaio and Varriale (2020) performed a single case study of blockchain applications in the aviation industry and found that blockchain-enabled information sharing did encourage trust and cooperation between airlines, airport operators, ground handlers, and air traffic controllers.

##### The execution of smart contracts and consensus mechanisms

Formalized arrangements of smart contracts and consensus mechanisms in the blockchain application also promote trust between supply chain parties (Palas & Bunduchi, 2020; Velmovitsky et al., 2021). The enforcement of a self-executing smart contract can ensure that transactions between two parties are governed and controlled by pre-agreed contracts and fines will be applied if one party exhibits opportunistic behaviour (Juma et al., 2019). Thus, trust bestowed from a trustor entity to other supply chain partners is expected to be improved (Baharmand et al., 2021; Qian & Papadonikolaki, 2020). For example, L'Hermitte and Nair (2020) argued that enhanced trust between participants stems from blockchain-enabled smart contracts that automate the resources matching and payment process as soon as pre-agreed conditions are met.

#### Consumers/public and a unit of supply chain pairs

The third pair of relationships belongs to the trustor of the two external stakeholders: consumers/the public and the trustee of the supply chain unit. In this regard, consumers and the public mainly evaluate the degree of trustworthiness of a supply chain through their perceptions of the trustee’s ability to commit to the delivery of desired end products (Sirdeshmukh et al., 2002). Such perception is dependent on information provided from the supply chain regarding products’ provenance, authenticity, custody, and integrity as well as production, modification, and supply chain protocol (Montecchi et al., 2019; Suhail et al., 2020).

Blockchain implementation comes into play by increasing the information authenticity and transparency of the supply chain which could lead to enhanced trustworthiness perceived by external stakeholders. This is mainly since the technology is able to promote information sharing from every supply chain member and tampers the risk of fraudulent practices such as counterfeit product and forgery reports (Garrard & Fielke, 2020; Kayikci et al., 2020). In other words, consumer and public acknowledgement regarding the material flow and product journey across the value chain are improved (Hasan et al., 2020; Köhler & Pizzol, 2020). For instance, Luzzani et al. (2021) empirically revealed that the blockchain-enabled collection of transparent information regarding soil and water features, climate condition, treatment with pesticides and fertilizers, production process, traceability, and labour and human rights helped enhance consumers’ trust placed on wine producers, and this was reflected in sales growth. Hence, it is expected that the trustor entity will have more confidence in supply chain commitment and bestow a higher level of trust to the supply chain showing quality data recorded for the whole supply chain process and activities (Cao et al., 2021; Li et al., 2020).

The enhancement of consumer trust is critically important in supply chains that require high quality and safety standards, such as food and pharmaceutical (e.g., X. Yang et al., 2021b; Yong et al., 2020), and supply chains that are subjected to asymmetric information such as second-hand markets (e.g., de Boissieu et al., 2021; Subramanian & Thampy, 2021). For instance, Yong et al. (2020) discussed how blockchain can help improve consumer and public trust in the vaccine distribution supply chain by tracking the flow of medicine along the supply chain using authentic drug identity and showing such information to consumers and the public. By the same token, the findings from previous literature also indicated that the employment of blockchain-predicated solutions helped increase brand reputation protection and customers’ trust establishment in the second-hand luxury market (de Boissieu et al., 2021) as well as the pre-owned, online electric-vehicle market (Subramanian & Thampy, 2021).

### Forms of trust

From the outcome of the deductive analysis using the three foundational categories of trust proposed by Rousseau et al. (1998) and Lewis and Weigert (1985), we found that the blockchain literature mainly discussed cognition-based trust (90 articles) and institution-based trust (48 papers), and rarely mentioned affect-based trust (nine articles).

#### Institution-based trust

The literature review revealed that institutional trust was mainly enhanced by the implementation of blockchain within pairs of trading partner relationships through smart contract applications and credit-based payment mechanisms (e.g., Palas & Bunduchi, 2020; Qian & Papadonikolaki, 2020).

*Smart contract applications.* Blockchain uses a consensus mechanism which all network participants need to agree to before executing the system (Cao et al., 2019; Pan et al., 2020). This means that when an issue arises, participants within the network can be confident that appropriate sanctions to rectify the issue will automatically be enforced through the smart contract (Chang & Chen, 2020; Pranto et al., 2021). With this effective control mechanism, the smart contract can help a trustor organization ensure that its trading partners within the supply network are discouraged from conducting opportunistic behaviours. Hence, the utilization of the blockchain platform is expected to increase the trustworthiness of a trustee party in performing trade with a trustor organization.

##### Credit-based payment mechanisms

Moreover, blockchain also facilitates effective payment mechanisms to prevent deferred payments (Qian & Papadonikolaki, 2020). As included in an agreed contract and also integrated with the tracking record, the payment protocol will automatically prevent the incidence of late payments (L'Hermitte & Nair, 2020; Tezel et al., 2021). Therefore, the blockchain-enabled, credit-based payment scheme can also fortify institution-based trust between supply chain members (Qian & Papadonikolaki, 2020; Viriyasitavat et al., 2019).

#### Cognition-based trust

The blockchain literature demonstrates that cognition-based trust is likely to be enhanced by the execution of the blockchain solution in all three pairs through the increase in information authenticity and transparency (Di Vaio & Varriale, 2020; Hasan et al., 2020; Juma et al., 2019). The superiority of data security and the consensus mechanism of blockchain execution facilitates the continual sharing of information from all relevant parties in the system (Kshetri, 2018), and it is reasonable to expect that a trustor will gain more knowledge of a trustee.

Considering each pair of relationships, cognitive trust is increased for system users when blockchain enhances the data integrity and authenticity of its equipped system (Cha et al., 2020; Juma et al., 2019). Similarly, the calculative trust between supply chain partners is expected to be substantively enhanced when the blockchain is presented, as the authenticity of essential business information of all relevant parties in the supply chain is verified and such information in the blockchain platform becomes visible to all supply chain parties (e.g., Centobelli et al., 2021; Di Vaio & Varriale, 2020; Wan et al., 2020).

For instance, Liu et al. (2021) explained that the transparent information system based on the blockchain can help address the lack of trust issue in financing between deep-tier retailers that are likely to be small and medium enterprises (SMEs) and commercial banks. This is mainly because blockchain increases visibility in SMEs’ credibility which leads to an increase in the cognitive trustworthiness that financial institutions place on SMEs.

In a similar fashion, the increased transparency and availability of information across the end-to-end supply chain also promotes consumers’ and the public’s cognition-based trust in the supply chain unit (e.g., Hasan et al., 2020; Rogerson & Parry, 2020). For example, blockchain can help inform consumers regarding the origin of products’ components and the production process of the finished products in the fishery supply chain, which consequently helps solve the trust crisis issue in fish consumption which mainly stems from a deficiency of information (Probst, 2020). Similarly, blockchain deployment can foster consumers’ trust and help address the issue of serious health and safety concerns in food supply chains during the COVID-19 pandemic through the reinforcement of information visibility (L. Yang et al., 2021a).

#### Affect-based trust

From our review of the existing literature, it can be said that blockchain may not have a direct impact on the enhancement of affect-based trust between organizations. For instance, Joo and Han (2021) stated that distributed trust along supply chains that was developed through blockchain, resembles the system-like trust (consists of reliability, functionality, and helpfulness) more than human-like trust (consists of integrity, ability, and benevolence). In addition, Wang et al., (2019a) highlighted that blockchain is more likely to instantly build digital trust rather than traditionally relational trust that takes years to build.

There are two reasons to support this:

1. As technology cannot prevent opportunistic behaviour and an organization’s misconduct in organizing physical information, technology is not able to promote emotional trust between two entities (Kopyto et al., 2020). Specifically, for supply chain partners, Schmidt and Wagner (2019) and Qian and Papadonikolaki (2020) argued that there is still a need to develop relational governance to facilitate affective trust in the long term, to maintain harmonious relationships between supply chain partners even though the blockchain is adopted.
2. For interorganizational trust between supply chain partners, several scholars stated that the employment of blockchain will shift the form of trust from affect-based trust to institutional and cognitive trust as technology facilitates the superiority of digital information security and transparency and enables credible control mechanisms (e.g., Schmidt & Wagner, 2019). It is reasonable to perceive that blockchain transforms and quantifies traditional, relational trust in the supply chain into a digital credit (Fu et al., 2020).

### Temporal orientation

From the outcome of deductive analysis using two predefined categories, the temporal dimension of trust is the least attention gained from blockchain scholars as only six papers investigated blockchain-enabled swift trust and another six papers implicitly discussed trust developed in the long term after implementing the technology.

#### Swift trust

From the six blockchain papers that discussed it, swift trust was found to be developed in the humanitarian supply chain where multiple aid agencies, emergency responders, and commercial organizations worked together to complete the specific, time-limited tasks of providing relief aid and minimizing suffering and death to those people affected by disasters (e.g., Dubey et al., 2020b; Hunt et al., 2021; Khan et al., 2021).

The execution of blockchain can enhance swift trust in the disaster-relief supply chain through three mechanisms. First, blockchain operation can increase ongoing information sharing and the visibility of secured, verified information which leads to the construction of rapid trust among various stakeholders in the supply network (Baharmand et al., 2021; Dubey et al., 2020b). For instance, Khan et al. (2021) found that transparency mediates the relationship between blockchain execution and trust performance in supply chains. Second, as the blockchain facilitates a real-time performance rating to each involved unit (a peer-rating system), accumulated rating scores can help indicate service users and providers’ capability and help establish a rapid trust among unknown actors (Hunt et al., 2021; L'Hermitte & Nair, 2020). Third, blockchain platforms help establish clear, consensual protocols and norms which help clarify roles and responsibilities and govern the actions of unknown users and providers of logistics resources (Hunt et al., 2021; L'Hermitte & Nair, 2020).

Notably, previous studies of swift trust mostly focused on supply network partner pairs and the forms of swift trust examined were mainly a combination of institution-based trust (protocols and rules) and cognition-based trust (knowledge from rating system and information sharing).

#### Long-term oriented trust

From the six papers that discussed the implication of blockchain on trust formation in the long term, there were two main streams of discussion: the possibility and issue of blockchain implication on trust between supply chain partners in the long term (Kopyto et al., 2020; Schmidt & Wagner, 2019), and the conjecture of users’ trust in the blockchain in the long term (Cha et al., 2020; Li et al., 2021b).

For supply chain partners, blockchain may not be able to mitigate opportunistic behaviours if there are no effective governance structures and active trust management between supply chain members (Kopyto et al., 2020; Schmidt & Wagner, 2019). Thus, affective trust may not be built due to the reduced honesty and integrity of each party (Sheppard & Sherman, 1998) and cognitive trust may deteriorate due to the negative perceptions of other partners (Doney & Cannon, 1997). Ultimately, blockchain alone may not be capable of building an ideal trusted ecosystem of supply chain networks. In the long run, other instruments and mechanisms are required to be involved in the sustainable enhancement of trust in supply chain management.

With regard to users’ trust in blockchain technology, Cha et al. (2020) emphasized the importance of blockchain, especially for long-lifecycle systems in securing the standard of data integrity, security, availability, and confidentiality in the long term. This helps stress the possibility that blockchain equipment can help facilitate system-associated trust in the long term if the technology still maintains its performance standards. In the same vein, Li et al., (2021a, 2021b) argued that blockchain needs to overcome the current security challenges of encryption algorithms and the consensus mechanisms which will enhance system security to make blockchain performance more rigorous and reliable at a future date.

### The literature summary and gap spotting

We summarize our discussions of blockchain and trust in supply chains grounded on the three trust dimensions in Table 5. The summary of trust discussion also highlights prominent gaps with respect to the various dimensions of trust and makes suggestions for future research accordingly.

**Table 5:** The roles of blockchain in facilitating trust in supply chains grounded on the three trust dimensions and identified research gaps

Trust dimension	The roles of blockchain	Research gaps
*I. Trustor–trustee* *Perspective*		
1. The users and blockchain pairs	Due to an increase in the capability of the blockchain to perform information storage and exchange, an organization as a user is likely to bestow a higher level of trust to the blockchain compared to traditional supply chain systems (Garrard & Fielke, 2020) Three sources of a trustee’s perceived higher capacity: (1) Blockchain’s superiority of security strength (e.g., Köhler & Pizzol, 2020; Musamih et al., 2021) (2) Blockchain’s faster speed of data processing and transmission speed (e.g., Palas & Bunduchi, 2020; Wang et al., 2019a, 2019b) (3) Blockchain’s self-executing governance and monitoring (e.g., Alkhoori et al., 2021; Qian & Papadonikolaki, 2020)	
2. The two supply chain partners pairs	There are two schools of thought, TTS and ITR, possessing different views on the implications of blockchains on the trust element between two supply chain partners. Actors who assume the roles of a trustor and trustee in this pair of relationships are also different	The dichotomy of views regarding blockchain implications for trust in supply chains could be an important issue for future investigation, especially the supply chain contexts which each view can be fitted into
2.1 TTS	In TTS, there is no need for interorganizational trust development between the two supply chain parties as they can both act as a trustor and bestow trust to the blockchain when performing exchanges (Kumar et al., 2020; Tezel et al., 2021) Put differently, blockchain solution plays a role as the trusted third party that facilitates transactions between supply chain members, thus trust from each member is redistributed and more centralized toward the operating system (Cha et al., 2020; Pournader et al., 2020b)	
2.2 ITR	In ITR, blockchain employment is expected to contribute to the reinforcement of interorganizational trust between supply chain partners (e.g., Dubey et al., 2020b; Surjandari et al., 2021) There are three primary arguments to support such trust enhancement: (1) Transference of the capabilities and strengthened trustworthiness in the blockchain to the organization that uses the system (e.g., Hunt et al., 2021) (2) Increase in the continuous exchange of information between two supply chain partners encouraged by the blockchain mechanism (e.g., Di Vaio & Varriale, 2020) (3) The facilitation of automated governance and monitoring of transactions performed using the application of smart contracts and consensus mechanisms (e.g., Palas & Bunduchi, 2020)	
Trustor: consumer and public Trustee: supply chain	Blockchain implementation helps strengthen the trust of consumers and the public through the enhancement of information authenticity and the transparency of various supply chain activities, the flow of materials and products along the supply chain, and the risks of supply chain breach or failure to perform (e.g., de Boissieu et al., 2021; Garrard & Fielke, 2020; Kayikci et al., 2020)	
*II. Form of Trust*		
1. Institution-based trust	The implementation of blockchain mainly bolsters institution-based trust between a user and the blockchain (e.g., Cao et al., 2021) as well as supply chain trading partners (e.g., Qian & Papadonikolaki, 2020) through: (1) The application of automated smart contracts ensure that sanctions will be incurred appropriately by parties responsible for misconduct; this discourages supply chain partners from engaging in opportunistic behaviours (e.g., Palas & Bunduchi, 2020; Pranto et al., 2021) (2) Credit-based payment mechanisms which help prevent the incidence of late payment through the self-executing tracking record (e.g., Qian & Papadonikolaki, 2020; Tezel et al., 2021)	
2. Cognition-based trust	Blockchain deployment is expected to increase cognition-based trust in each of the three pairs of relationships through the following mechanisms: (1) For trust in the operating system, blockchain integration can promote users’ cognitive trust through the enhancement of data integrity and authenticity (Cha et al., 2020; Juma et al., 2019) (2) For supply chain partners, blockchain adoption can reinforce cognition-based trust through the encouragement of credible information sharing and the visibility of real-time updated information (e.g., Liu et al., 2021; Wan et al., 2020) (3) For the trust of consumers/public in a supply chain unit, blockchain implementation can strengthen the cognition-based trust in the supply chain through the authenticity and transparency of information regarding various activities end-to-end across the supply chain (e.g., Rogerson & Parry, 2020; Yong et al., 2020)	
3. Affect-based trust	Blockchain employment may not have a direct influence on affect-based trust between supply chain partners for two reasons: (1) If not equipped with suitable governance mechanisms, the technology alone may not be able to prevent the opportunistic behaviour of a supply chain partner in organizing physical information (Kopyto et al., 2020) (2) Affect-based trust may be quantified and transformed into cognition-based trust in the form of digital credit (Qian & Papadonikolaki, 2020)	More conceptual and empirical research is needed to examine affect-based trust and blockchain adoption in various settings
*III. Time Dimension*		
1. Swift trust	The blockchain shows the potential to increase swift trust in disaster-relief supply chains through the three following mechanisms: (1) Encouragement of ongoing information sharing between unknown partners and enhancement of information visibility and transparency end-to-end throughout supply chains (Baharmand et al., 2021; Dubey et al., 2020b; Khan et al., 2021) (2) Provision of the real-time performance rating system which allows unknown partners to rapidly discern the capability of others to perform (Hunt et al., 2021; L'Hermitte & Nair, 2020) (3) Clear and consensual protocols for governance actions of unknown supply chain partners (Hunt et al., 2021; L'Hermitte & Nair, 2020)	There is a possibility that a blockchain can assume the role of a swift trust facilitator in other contexts apart from disaster-relief supply chains
2. Long-term oriented trust	Blockchain implications for long-term oriented trust is only discussed in the trust between two supply chain partners and the user’s trust in the technology-based system (1) As blockchain may not be able to reduce the opportunistic behaviours of trading partners over the long term when proper governance and active trust management are absent, the technology may have a minimal effect on trust formation for supply chain partners (Kopyto et al., 2020; Schmidt & Wagner, 2019) (2) For system users, blockchain is promising in its enhancement of the trustworthiness of its equipped system in the long term if the technology can maintain its standards of information-related capabilities and overcome the challenges associated with encryption algorithms and consensus mechanisms (Cha et al., 2020; Z. Li et al., 2021b)	More conceptual and empirical studies are needed to refine the concept and investigate how different forms of trust are affected by the presence of blockchain technology over a longer time period

### The blockchain-entrusted supply chain models

From the summary of the existing literature review, we can see that there are multiconnections between categories within the three trust dimensions. Therefore, in order to visualize all such linkages between various trust elements in the three proposed dimensions and consolidate the findings of this literature-based study, we developed a conceptual model that illustrates the formation of trust when implementing blockchain technology and the diffusion of trust through different components in the ecosystem of the supply chain.

**Fig. 1:**
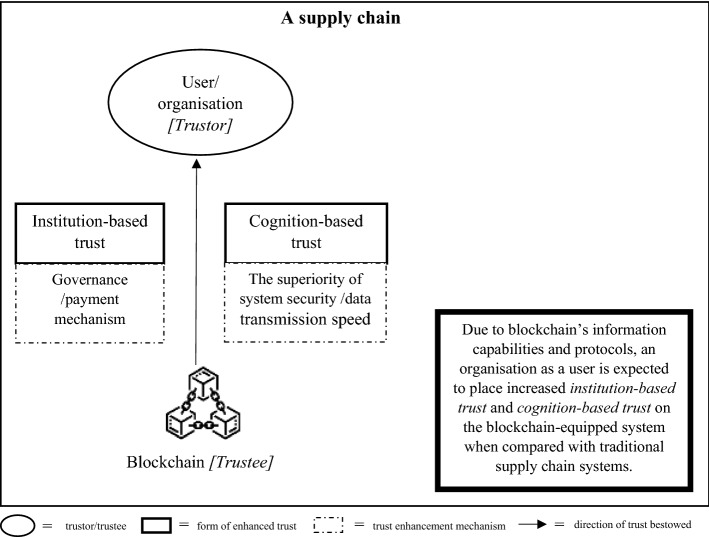
The enhancement of the organization’s trust in the blockchain in the Trustless trusted scheme (TTS) model

**Fig. 2:**
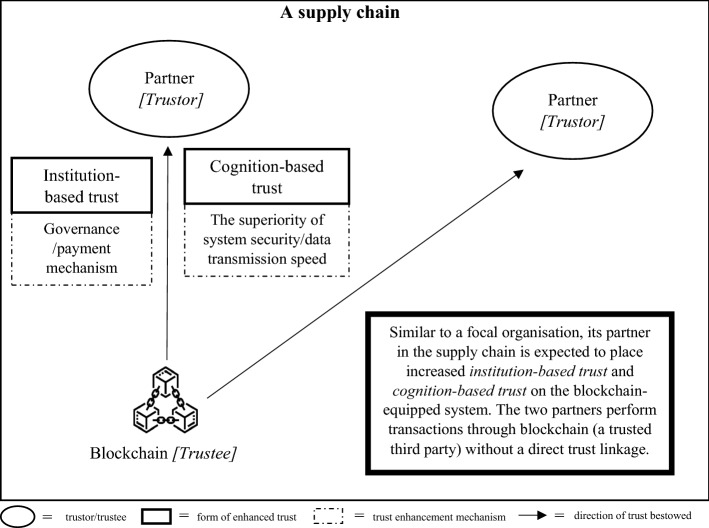
The impact of blockchain on trust between a pair of supply chain partners in the Trustless trusted scheme (TTS) model

**Fig. 3:**
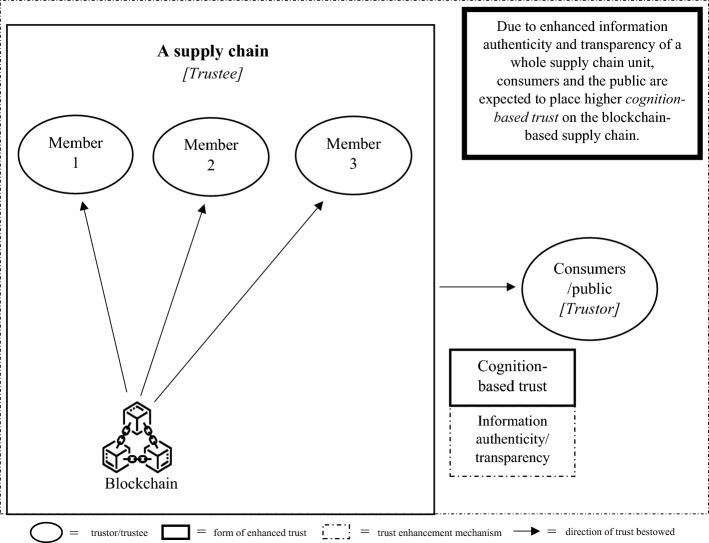
The trust enhancement of consumer and public in the blockchain-entrusted supply chain in the Trustless trusted scheme (TTS) model

**Fig. 4:**
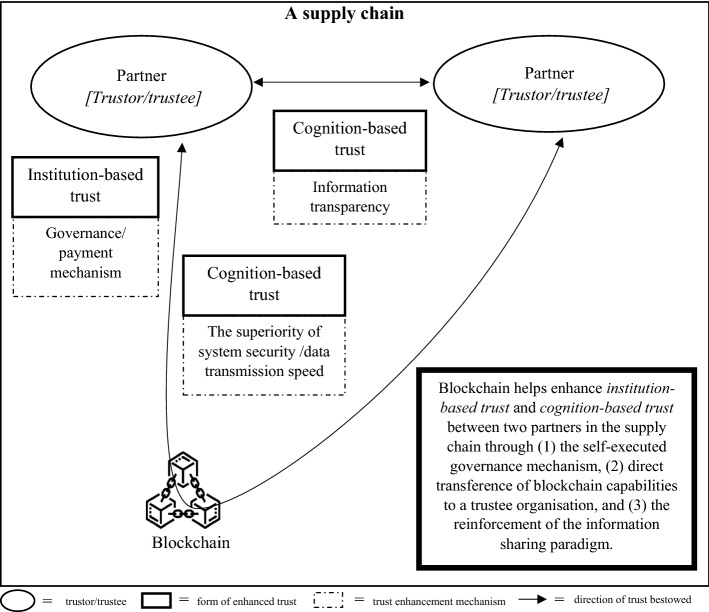
The impact of blockchain on trust between a pair of supply chain partners in the Interorganizational trust reinforcement (ITR) model

**Fig. 5:**
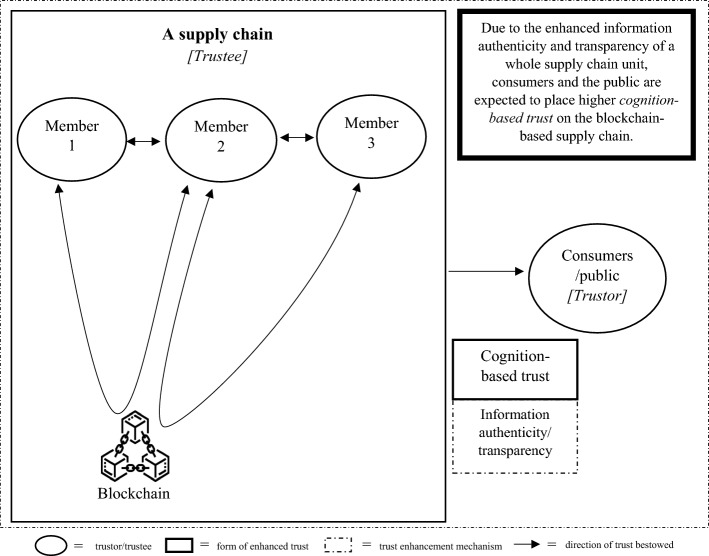
The trust enhancement of consumers and the public in the blockchain-entrusted supply chain in the Interorganizational trust reinforcement (ITR) model

As there are two schools of thought regarding the effect of trust on supply chain partners, we developed two different trust development models, called TTS and ITR models, with each model comprising three connected steps. Each step of each model portrays the implication of blockchain on a pair of trustors and trustees. In both versions of the proposed models, in the first step, we started from a pair of technology users and the blockchain to illustrate the direct impact of blockchain adoption on origination that uses the technology, as shown in Fig. 1. In the second step of both models, we illustrate the blockchain implications on the trust element between two supply chain partners, as presented in Fig. 2 for the TTS model and Fig. 4 for the ITR model. In this step, the two versions display the main differences in the impact of blockchains and actors who assume the roles of trustors and trustees in both the TTS and ITR models. In the third step, we completed the blockchain-enabled trust diffusion model with an increase in trust of a supply chain unit by the external stakeholder viewpoints as illustrated in Fig. 3 for the TTS model and Fig. 5 for the ITR model.

**Fig. 6:**
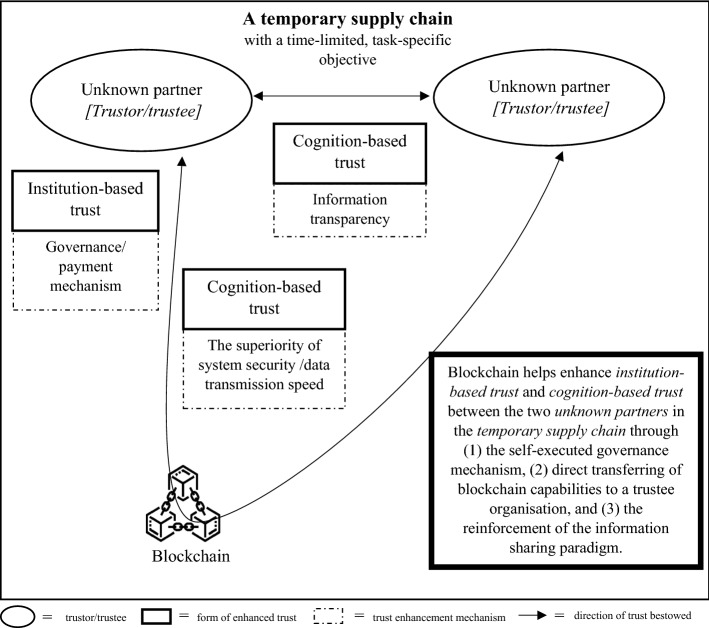
The development of the swift trust between unknown supply chain partners in a temporary supply chain in the swift trust development (STD) model

The three steps of trust development and dissemination start from the direct impacts of blockchain on its users, move to other supply chain members in a subsequent step and end with the extent of impact on a whole supply chain unit from the eyes of external consumers and public. This approach delicately reflects the step-by-step ramification of blockchain adoption on trust subject within a range of stakeholder entities involved in the supply chain ecosystem. Different forms of trust are also incorporated to help explain trust bestow between each trustor–trustee pair. Moreover, to clearly show the development of swift trust between supply chain partners under certain circumstances, we developed a separate three-step model of swift trust development (STD). From our analysis of the literature, it is likely that the STD model is the emulation of the ITR model with modified components in step two, as shown in Fig. 6.

#### The TTS model of blockchain-entrusted supply chains

The first step of the TTS model expounds trust in a pair of blockchains and users. As shown in Fig. 1, the adoption of a blockchain instead of a traditional operating system in the supply chain is expected to increase institution-based trust (e.g., Qian & Papadonikolaki, 2020; Tezel et al., 2021) and cognition-based trust (e.g., Alkhader et al., 2021; Kayikci et al., 2020) in a system user or an organization that operates the technology. An increase in institutional trust is the outcome of a pre-agreed governance protocol and payment mechanism which will be self-executed when operating the blockchain (Qian & Papadonikolaki, 2020). As the blockchain as a trustee entity demonstrates such an effective governance mechanism in performing transactions, an organization is believed to attribute the trustee with a higher capability to complete an exchange and bestow a higher trust to the system (e.g., Asante et al., 2021; Palas & Bunduchi, 2020).

Increased cognition-based trust mainly stems from the two capabilities of blockchain to enhance system security strength and speed of data transmission which collectively create the visibility of nearly real-time, credible information (e.g., Hu et al., 2021; Viriyasitavat et al., 2019). As there is more authentic information available, a trustee will have more knowledge regarding the trustee’s enhanced capability. Therefore, an organization operating the blockchain as a trustor entity is expected to bestow higher trust to the supply chain system equipped with blockchain.

The second step of the TTS model provides the delineation of the impact of blockchain on trust between a pair of trading partners. As scholars believe that blockchain implementation eliminates the need for developing trust between the two organizations (e.g., Kumar et al., 2020; Pournader et al., 2020b), there is no direct trust linkage from one organization to the other, as shown in Fig. 2. Moreover, as the TTS intellectual community believes that the blockchain acts as a trusted third party and redistributes the trust of two supply chain partners towards itself, there are two straight lines connecting the two parties to the blockchain. As in step one, as the blockchain solution shows an increase in capability to store and exchange information when organizations perform their transactions, the two organizations as trustors are likely to provide more trust to the blockchain compared to traditional supply chain systems. The forms of trust are also identical to those in the first step: institution-based and cognition-based trust. The logic as to why two such forms of trust are enhanced also resembles the first step with the duplication of two organizations, thus there is no further explanation provided in this step.

The third step in the TTS model delineates the trust enhancement of an end-to-end supply chain from the view of external consumers and the public, as shown in Fig. 3. Due to blockchain-enabled superior security of information sharing and the faster speed of information transmission, every supply chain member is expected to exchange authentic and credible information through the third-party blockchain solution in a hypothetically real-time fashion (Garrard & Fielke, 2020; Kayikci et al., 2020). Therefore, information authenticity and transparency of a whole supply chain unit are likely to be enhanced, leading to more credible knowledge provided to external stakeholders (Subramanian & Thampy, 2021). It is then logical to expect that consumers and the public will gain confidence and provide more trustworthiness to the supply chain entrusted with blockchain (Musamih et al., 2021; Pawar et al., 2020; Yong et al., 2020).

#### The ITR model of blockchain-entrusted supply chain

As the first step of the model only shows the trust relationship between the blockchain and its user, the logical explanation of the direct blockchain impact according to distinct features of the technology in the ITR model is identical to the TTS model. Therefore, no further explanation is provided in this step. In ITR intellectual commitment, scholars believe that the presence of blockchain helps enhance trust bestowed between a pair of supply chain partners through three complementary mechanisms, namely: (1) the self-executed governance mechanism, (2) direct transferring of blockchain capabilities to a trustee organization, and (3) the reinforcement of the information sharing paradigm. As presented in Fig. 4, there are two lines of trust connection: the curved line that passes through a blockchain and the straight line that directly connects two supply chain partners. The curved line represents mechanisms of trust creation (1) and (2), in which trust is enhanced in the form of institution-based trust and cognition-based trust, respectively.

##### Increased institutional trust in the curved line

Similar to the TTS model, the blockchain application of smart contracts enables an effective mechanism to govern a transaction between two parties, thus increasing the level of institution-based trust to both supply chain partners (e.g., Palas & Bunduchi, 2020; Qian & Papadonikolaki, 2020). This effective governance capability is transferred from blockchain intrinsic features to the trustee organization that operates the technology.

##### Increased cognition-based trust in the curved line

As a trustee organization provides more credible, nearly real-time information when equipped with the blockchain, it is reasonable to expect that a trustor organization will have more knowledge of the trustee and bestow a higher level of cognitive trust (K. Li et al., 2021a). Such enhancement in credibility and speed of information communication stems from the transference of the blockchain’s superior capability of security systems and data transmission speed (Palas & Bunduchi, 2020).

##### Increased cognition-based trust in the straight line

Since the information in blockchain is safely secured and there are appropriate, pre-agreed sanctions for those who perform information misconduct, the presence of the blockchain encourages the two supply chain parties to continuously share their business information with each other (e.g., Ghode et al., 2020; Li et al., 2020). The enhancement of information sharing leads to the establishment of an information transparency paradigm and increases the trustor’s knowledge regarding the trustee, thus there is likely to be more trust exchanged between the two partners (e.g., Pan et al., 2020).

As in the TTS model, the third step of the ITR model portrays the perceived increase in cognition-based trust of a supply chain unit from the eyes of external consumers and the public, as shown in Fig. 5. Since the constellation of (1) reinforcement of information sharing between multiple supply chain members, (2) superior security of information flowing end-to-end along the supply chain, (3) faster speed of information communication between multiple pairs of participants, and (4) self-executing governance and payment mechanisms that apply to all pairs of supply chain partners, it is reasonable to expect a tremendous increase in information authenticity and the transparency of the whole supply chain. Hence, external stakeholders, consumers, and the public will be likely to gain more credible knowledge regarding the multitude of activities inside the supply chain, leading to an enhancement of confidence and greater trust in a supply chain with blockchain (Hasan et al., 2020; Köhler & Pizzol, 2020; Lee & Yeon, 2021).

#### The STD model of blockchain-entrusted supply chain

Again, since the first step of the swift trust model starts from the direct impact of blockchain adoption on its users’ trust, the rationale behind the trust development is the resemblance of step one in TTS and ITR models. Therefore, no further explanation is provided in this step. From the body of literature reviewed, the mechanisms of swift trust building in the second step are identical to that of the ITR model. Therefore, we developed this step of the STD model by emulating the second step of the ITR model and modifying essential components, as shown in Fig. 6.

Specifically, we changed a unit of *supply chain* in the ITR model to *temporary supply chain*, which serves task-specific objectives and will last for a short time period. This temporary supply chain is typically found in the humanitarian supply chain, which prior studies have found appropriate for swift trust-building (Dubey et al., 2020b; Hunt et al., 2021; L'Hermitte & Nair, 2020). Furthermore, we changed from *partner* in the ITR model to *unknown partner* in the STD model, as swift trust is hypothetically built between two organizations that have never worked together before (Dubey et al., 2019). Since the logic behind the enhancement of trust between unknown partners from blockchain in swift trust resembles the second step of the ITR model, no further explanation is provided in this section. It is also crucial to mention that there is no study indicating the implication of blockchain on swift trust using the mechanism of the TTS model.

From the result of the literature review, the discussion about the blockchain implication on the development of the swift trust in the whole supply chain unit is limited. However, since the mechanism of swift trust formation in the second step is identical to that in the ITR model, we can expect the constellation of four trust enhancement mechanisms which lead to an increase in information authenticity and transparency of an end-to-tend temporary supply chain. External stakeholders of a temporary supply chain then gain more credible knowledge of activities inside, hence they are likely to bestow higher trust to the blockchain-entrusted supply chain.

### Further discussions on the proposed TTS, ITR, and STD models

After exploring the TTS, ITR, and STD models in detail, we can see that there are several commonalities of blockchain impact and trust development in the three models. Nevertheless, there are also the main components in each model that disparate one from the others. We, therefore, summarize commonalities and differences in this section.

First, the three models share the same trust developmental process in step one which describes the direct impact of the blockchain on an organization operating the technology. In other words, the adoption of blockchain technology is the absolute origin of trust dissemination in the supply chain ecosystem. Second, although the mechanisms of trust development inside a supply chain in the three models are heterogeneous, the blockchain-entrusted supply chain is likely to enhance external consumer and public trust in a whole supply chain unit. Third, institutional-based trust, as well as cognition-based trust, are the two forms of trust that contribute to the trust development and diffusion in all three models.

The main differences between the three models lie in the second step of the model where there is trust enhancement between two supply chain partners with the presence of a blockchain. Specifically, for the TTS model, there is no direct linkage between two supply chain parties, yet the two organizations act as trustors who bestow trust to the blockchain (e.g., Cocco et al., 2021; Kumar et al., 2020). In this scenario, the blockchain plays the role of a trustee who shows a higher capability to perform transactions with the superiority of system security and information transmission speed (e.g., Palas & Bunduchi, 2020; Viriyasitavat et al., 2019). In contrast, in the ITR and STD models, the blockchain acts as a facilitator that enables trust development between the two supply chain parties. In this scenario, the two parties both act as a trustor who renders trust and a trustee who is perceived as having greater trustworthiness. Such an increase in the trustworthiness of a trustee party comes from two mechanisms. First, the capabilities of blockchain regarding such superiority of system security and information communication speed are transferred into an organization operating the technology (e.g., K. Li et al., 2021a; Shahid et al., 2020). Also, the implementation of blockchain encourages two supply chain parties to exchange information, leading to the establishment of an information transparency paradigm (e.g., Ghode et al., 2020; Li et al., 2020).

With regard to the main differences between the ITR and STD models, step two of the STD model is a modified version of that in the ITR model. Specifically, the STD model presents a particular context in which swift trust can be developed with the presence of blockchain: a short-lived, temporary supply chain with task-specific objectives and trust exchanged between unknown partners (Dubey et al., 2020b; Hunt et al., 2021). In this scenario, both institution-based trust and cognition-based trust are expected to develop swiftly and only last for a short period of time. The summary of commonalities and differences of the three proposed models are shown in Table 6.

**Table 6:** Commonalities and differences of the TTS, ITR, and STD models

Model	Commonalities	Differences
TTS	Blockchain is an absolute origin of trust diffusion. The first step in the three models shares the identical trust enhancement mechanism A supply chain equipped with blockchain is likely to enhance external consumer and public trust Two forms of trust enhanced in the three models are institutional-based trust as well as cognition-based trust	In step two: No direct linkage between two supply chain parties The blockchain act as the third party when two parties perform an exchange
ITR		In step two: The blockchain acts as a facilitator that enables trust development between the two supply chain parties Increase in trust exchange between the two parties comes from the transfer of blockchain security and information communication capabilities as well as the establishment of an information transparency paradigm
STD		In step two: Same trust enhancement mechanism as in the ITR model Modify two components of the ITR model to a temporary supply chain and trust exchanged between unknown partners

## Discussion

In this literature-based review, we used the three dimensions of trust adopted from management research as a theoretical background to frame the content analysis and we used the perceived trustee’s capability to fulfill obligations from the eyes of the trustor to explain how such capability is enhanced by the presence of blockchain. The blockchain-entrusted model was then developed in the three variations to converge the contradicting views of blockchain application to trust in supply chains as well as present a case for swift trust formation mechanisms between unknown partners within supply chains. The main contributions of this study to theory and practice are discussed below.

### Theoretical implications

This study has been the first attempt to consolidate the body of knowledge addressing blockchain and trust in supply chains. Based on the three dimensions of trust adopted from management literature, the concept of trust in the supply chains adopting blockchain technology is elaborated. Using inductive and deductive approaches to review the extant literature, we classified trust in supply chains into categories according to the trustor–trustee perspective, forms of enhanced trust, and temporal orientation. For the trustor–trustee perspective, the outcome of the inductive content analysis revealed three pairs of trustor and trustee involved in supply chains using blockchains, i.e., a user and the blockchain, two supply chain partners, and consumer/public and a supply chain unit. The paired perspective indicated how blockchain capabilities facilitated the trustees’ capabilities in fulfilling obligations from the perception of trustors in each paired relationship. Likewise, the two subthemes under the two supply chain partners, namely TTS and ITR, emerged as contrasting views. These two views propose rather contradicting ideas on blockchain implications for trust in supply chains and the actors who assume the roles of trustor and trustee. Additionally, for the forms of trust, the outcome of deductive analysis using three rudimentary categories from management scholarship indicated that cognition-based and institution-based trust were likely to be enhanced by blockchain implementation, while affect-based trust, which is typically considered as traditional interorganizational trust, was not directly impacted by the technology. This dimension clarified how utilizing advanced technologies such as blockchain in supply chain settings further enhanced trust between two supply chain partners even though those technologies had a minimal effect on traditional trust between partners, characterized by bonds and attachments (Wong et al., 2008).

With respect to time orientation, the outcome of the deductive analysis through the categorization of swift trust and long-term oriented trust illustrated that swift trust increased between unknown supply chain partners under certain circumstances. Although the existing literature merely showed the potential of blockchain on swift trust formation in the humanitarian supply chain, the concept of blockchain-enabled swift trust can be applied to other supply chain settings in which two or more unknown partners need to be coordinated in a short-lived period to fulfill immediate goals. We subsequently developed the three models of blockchain-entrusted supply chains that showed the multiconnectivity of multiple aspects of trust enhanced by blockchain. The proposed model discussed three connected steps to initially develop trust through the implementation of blockchain that were subsequently diffused to supply chain partners and external stakeholders. The three variations of the model also discussed the TTS and ITR schools of thought as well as the STD model, which portrays the swift trust development in a temporary supply chain. Though these three models shared similarities, the review of the literature revealed that in the TTS model, trust was redistributed from supply chain partners to the blockchain as the third-party intermediary. This is somewhat in contrast with the ITR model, where trust between supply chain partners was enhanced through blockchain.

Our explanation of how blockchain is able to engender trust between supply chain participants also responds to the question raised by Böckel et al. (2020): “the question remains how a technology might be able to create trust, if there is a lack of trust in the first place” (p. 536). By using the TTS model, supply chain entities can perform transactions through the trusted blockchain as a third party. This means that they can only trust blockchain and information exchanged in the platform, not necessarily each other. In other words, as the two supply chain partners can trust blockchain and perform trusted transactions through the blockchain platform, there is no need for any type of trust to exist in the first place. The development of the TTS model also responds to the concern raised by Caldarelli et al. (2020): “in the early literature, the blockchain was viewed as a means for creating trust, while it is now widely held that blockchain provides a way to transact in a trustless environment” (p. 10). In the TTS model, blockchain as a trusted third party does create trust between a pair of supply chain parties and the blockchain system, and also enables a trustless scheme of transactions performed among supply chain participants.

In addition, the contrasting views of the TTS and ITR models lay the foundation for future studies to validate these models through the empirical investigation of a variety of supply chains. Both models could be valid to explain practical blockchain use cases, yet each of them would only suit certain supply chain contexts. In particular, given the different trust formation and diffusion mechanisms in the two models, a variation in the *initial trust condition* of participants in different supply chain contexts would affect the model’s suitability. Finally, the swift trust creation mechanisms illustrated in the STD model also provides the basis for further research to verify this model in other contexts apart from humanitarian supply chains. Although existing literature has developed the concept of swift trust from humanitarian contexts, some certain conditions of the STD model, i.e., temporary settings and unknown partners, could be applied to other supply chain settings.

### Managerial implications

The outcomes of this study can help corroborate the impact of blockchain implementation on trust in supply chains. The study can support an organization’s decision to adopt blockchain technology for managing its supply chain, particularly when supply chain operations are disrupted, such as is the case with COVID-19, when high levels of trust are required in supply chains to manage disruptions. According to a survey of more than 600 procurement, supply chain and business leaders, Procurious (2020) found that the COVID-19 crisis caused 65% of respondents’ firms to not have confidence in their current suppliers and to prepare alternative sources of supplies as they had insufficient information regarding those suppliers and their upstream supply chain. Similarly, the Lloyd’s Register’s survey of 100 senior executives in the beverage industry found that due to an increase in food fraud after the COVID-19 outbreak, only 22% of respondents showed confidence that their suppliers had met food safety standards (Berry, 2021). In these kinds of trust-disruption situations, the adoption of blockchain may be a promising solution for an organization in disrupted supply chains to regain trust in their supply chain partners. By adopting either the TTS or ITR models, supply chain partners benefit from the enhanced institution-based and cognition-based trust enabled by blockchain. The concept of swift trust-building and the STD model can also be practised for rapid trust-building between supply chain partners who might not be known to each other, especially during times of crisis. Trust is a core component in crisis management and blockchain promises to build such trust in a timely manner through the STD model. To summarize, blockchain technology is not only capable of providing a remedy for managing supply chain disruptions through trust, it can also mitigate trust-related disruptions.

In addition, the three blockchain-enabled trust models could be practically adopted in various supply chain contexts to address the long-standing trust issue between participating organizations. For instance, the TTS and ITR models could be adopted in food and pharmaceutical supply chains to address several trust-associated concerns such as food safety in cross-border food trade (ESCAP, 2018), the authenticity of halal-labelled food (Johari, 2016), and the standards of vaccine distribution (Woodley, 2019). With blockchain execution, cognition, and institution-based trust placed in the information exchanged, in other supply chain participants, and in the whole supply chain system are expected to be enhanced. Therefore, real-world trust issues revolving around product safety and standards could be alleviated by the utilization of blockchain (Alkhoori et al., 2021; Qian et al., 2020; Surjandari et al., 2021). Likewise, the adoption of either the TTS or ITR model in multilevel supply chains could also resolve the persistent problem of a lack of trust in deep-tier SMEs financing (Stolberg-Larsen, 2019). Since blockchain enables information visibility, SMEs’ credibility is enhanced, and the cognition trust from commercial banks is expected to increase (Liu et al., 2021). With such increased trust, the deep-tier SMEs could find it more manageable to seek loans from financial institutions with the presence of blockchain. Besides this, the TTS and ITR models could also be applied to address chronic trust-related problems arising from asymmetric information in second-hand markets (Jackson, 2020). As information and knowledge are more transparent due to blockchain capabilities, the buyer–seller information asymmetry is therefore mitigated. The buyers’ trust placed in second-hand products is then projected to increase (de Boissieu et al., 2021; Subramanian & Thampy, 2021).

## Conclusion

This review aimed to serve as the first comprehensive study that synthesizes and consolidates prior studies of blockchain and trust to answer the question of how this technology can impact the trust element in supply chains. The paper used the three dimensions of trust adopted from management research to provide a framework for the content analysis and model building. With the combination of the inductive and deductive approaches, we extracted 94 selected publications in top-tier journals, published between 2018 and 2021. We then developed a blockchain-entrusted model showing the interconnection of the three trust dimensions based on the result of content analysis. The main findings of the study highlight the contradicting assertions among scholars on the implications of blockchain for trust in supply chains (Shao et al., 2022). While some studies pointed out that blockchain would enable a trustless scheme, others expected the reinforcement of interorganizational trust. The blockchain-entrusted supply chain models are developed in three variations to conceptualize these assertions as well as a case for swift trust formation under certain circumstances in supply chains. The proposed models also present the three-step process of how trust is enhanced through the blockchain and diffused to supply chain partners and external stakeholders. The outcome of this literature synthesis and the proposed blockchain-entrusted models helped identify several gaps in the extant literature and lay foundations for future research implications as follows.

First*,* future studies need to empirically examine the contradicting views expressed by scholars with respect to TTS and ITR models. At this stage, considering that there is a limited number of blockchain practical use cases owning to a very early stage of the blockchain applications for supply chains, we cannot find an appropriate example of actual blockchain use cases that suits the TTS and ITR models. Nevertheless, we conjectured that the two models would fit with different contexts of supply chains. Given a difference in the initial trust condition of participants, both TTS and ITR would be only appropriate to certain supply chain contexts. Specifically, the TTS model is suitable for explaining blockchain implementation in the *low trust climate supply chains*, while the ITR model is suitable for blockchain implementation in the *high trust climate supply chains.*

For the TTS model, the existing literature suggested that the key strengths of blockchain that enable the trusted exchange of data directly without an intermediary make the technology more suitable for low-trust ecosystems (e.g., Hijazi et al., 2021; Longo et al., 2019). Kumar et al. (2020) stated that in a comparison of blockchain and other interorganizational technologies, the disseminated power of blockchain infrastructure that reduces the need for trust among supply chain actors and for an intermediary leads to the suitability of blockchain in low-trust climate supply chains, while in supply chains where there is high trust among parties and low traceability and visibility required, conventional technologies such as electronic data interchange would be more appropriate.

For the ITR model, the existing literature suggested that blockchain-enabled information transparency and traceability may help maintain high levels of trust among participants in supply chains where trust has already been built over years (Caldarelli et al., 2020). This means that blockchain would not be a “trust creator” developing interorganizational trust from zero, yet the technology would play a role as “trust maintainer/sustainer,” which works well in an environment where trust is at a maximum by providing defensive mechanisms against external threats such as counterfeit products (Caldarelli et al., 2020; Mendling et al., 2018). As blockchain is capable of enhancing cognition and institution-based trust, the role of blockchain as a trust sustainer in the supply chains exhibiting a high trust establishment among supply chain actors is then well-grounded by the explanation that blockchain transforms such long-established, interorganizational trust (likely to be affect-based trust) into cognition and institutional trust, and maintains the overall level of trust among participants.

Nonetheless, these conjectures about the contextual suitability of our proposed models still need further verification. We then call for future research to test the conceptual model of blockchain-enabled trust in multiple supply chain contexts.

Second*,* the investigation of swift trust application to supply chains beyond studies surrounding humanitarian supply chains is required. Currently, the study of swift trust outside the humanitarian supply chain is very limited, with the possibility of future disruption in multiple supply chains, the formation of swift trust in other contexts may be worth investigating (Casady & Baxter, 2020). For instance, with the widespread interruptions of COVID-19, many supply chains experienced disastrous impacts from disruption in their upper-tier suppliers. Downstream buyers then needed to source raw materials from new suppliers that they might not have worked with before (Kilpatrick, 2020). In the situation where downstream purchasers are required to form a short-lived relationship with unknown suppliers, the use of blockchain technology could help create swift trust and facilitate the completion of material delivery during a time of crisis.

Finally, although current studies argue that blockchains might not be directly linked to enhanced affect-based trust in supply chain relationships, they still suggested that the technology may have an indirect, positive impact on the reinforcement of relational trust between supply chain partners through the facilitation of cognition and institutional trust (Schmidt & Wagner, 2019). Future investigations on blockchain and the trust formation process between supply chain partners would provide insightful information on the dynamic formation of trust and the role of blockchain technology in facilitating such a process in the long term.

## Supplementary Information

Below is the link to the electronic supplementary material.

Supplementary file1 (DOCX 101 KB)
