# Supplementary material for: Blockchain as the “trust-building machine” for supply chain management
Source: Ann Oper Res. 2022 Aug 5:1–40. Online ahead of print. doi: 10.1007/s10479-022-04868-0 (PMC9362671; doi:10.1007/s10479-022-04868-0)
Supplement: Supplementary file 1 — Supplementary file1 (DOCX 101 KB) [file 10479_2022_4868_MOESM1_ESM.docx]

# **Online Appendix A. Summary and categorisation of the extant literature on supply chain, blockchain and trust**

| **Articles** | **Summary** | **Trustor-trustee Perspective** | **Form of Trust** | **Time Orientation** |
| --- | --- | --- | --- | --- |
| Meng, Tischhauser, Wang, Wang, and Han (2018) | The conceptual paper discussed the applicability of blockchain technology to resolve trust management issues within the current detection system architectures. Specifically, the blockchain-based approach enables trust computation which utilises alert information to evaluate the trustiness of other parties. The findings highlighted that blockchain-integrated architecture has the potentials to improve the performance of intrusion detection systems. | 1. The pair of users and blockchain-based system  2. The pair of two Supply Chain Partners - Trustless trusted scheme (TTS) | 1. Institution-based Trust  2. Cognition-based Trust | - |
| Pärssinen, Kotila, Rumin, Phansalkar, and Manner (2018) | The article presented the way in which blockchain can revolutionise the online advertising supply chain by shifting the paradigm of trust and authenticity. Specifically, the protocols of blockchain create trustless systems where there is no need for third-party verification of any transaction transpired. | 1. The pair of users and blockchain-based system  2. The pair of two Supply Chain Partners - Trustless trusted scheme (TTS) | 1. Institution-based Trust  2. Cognition-based Trust | - |
| Benčić, Skočir, and Žarko (2019) | The paper proposed a blockchain and Internet of Things (IoT) integrated solution for the supply chain with a public ledger in a trustless environment. When connecting information from smart tags (set up by IoT) of each product to the distributed ledger technology, all relevant stakeholders are enabled to trace the products along their lifecycle and validate a product that they are handling and exchanging. This mechanism increases the authenticity of product data, which in turn enhance trust in the products without requiring a product consumer and relevant stakeholders to trust the intermediary platforms or other supply chain stakeholders. | 1. The pair of users and blockchain-based system  2. The pair of two Supply Chain Partners - Trustless trusted scheme (TTS) | Cognition-based Trust | - |
| B. Cao et al. (2019) | The conceptual paper briefly explained how blockchain and IoT can address security and trust concerns and collectively create a trusted platform for relevant stakeholders. The article also elaborated why a consensus mechanism is indispensable for blockchain and IoT integrated systems as well as demonstrated the utilisation of the two consensus mechanisms. | 1. The pair of users and blockchain-based system  2. The pair of two Supply Chain Partners - Trustless trusted scheme (TTS) | 1. Institution-based Trust  2. Cognition-based Trust | - |
| H. Hasan, AlHadhrami, AlDhaheri, Salah, and Jayaraman (2019) | The paper discussed how blockchain solutions can be applied to create efficient shipment management. The article highlighted that as a peer-to-peer, secured, distributed ledger, the blockchain-based platform eliminates the need for trusted third parties as intermediaries. By using smart contracts, both senders and receivers can perform the self-check of real-time trusted information whether the package is shipped as expected or not. | 1. The pair of users and blockchain-based system  2. The pair of two Supply Chain Partners - Trustless trusted scheme (TTS) | 1. Institution-based Trust  2. Cognition-based Trust | - |
| Juma, Shaalan, and Kamel (2019) | The conceptual paper outlined an overview of the blockchain application in international trade supply chains. The article highlighted the role of distributed ledger technology in establishing a secure communication paradigm between supply chain partners in which data integrity and immutability can be guaranteed.  Trust is also mentioned as one of the promising capabilities of blockchain technology as the characteristic of blockchain enables all participants within the network to have the same authority privilege to not control the system. In other words, the decentralised nature of blockchain helps increase trust among supply chain members. Also, the designed consortium mechanism based on the blockchain features helps increase trust between different parties. | 1. The pair of users and blockchain-based system  2. The pair of two Supply Chain Partners - Inter-organisational trust reinforcement (ITR) | 1. Institution-based Trust  2. Cognition-based Trust | - |
| Longo, Nicoletti, Padovano, d'Atri, and Forte (2019) | The study conducted an experiment of the blockchain-based supply chain simulation model to present the benefits of blockchain in overcoming trust and collaboration issues among supply chain partners and discouraging enterprises from misconduct such as counterfeiting data. The authors concluded that a blockchain-based system is expected to be very useful in low-trust environments or in situations that companies are not willing to share their confidential data with other supply chain members. | The pair of two Supply Chain Partners - Trustless trusted scheme (TTS) | Cognition-based Trust | - |
| Montecchi, Plangger, and Etter (2019) | The paper developed the conceptual framework to guide how blockchain application for supply chain management can increase consumers’ knowledge and assurances of products’ provenance and reduce several types of perceived risks. The findings suggested that when adopting blockchain, the provenance information is made available. The consumer trust is then reinforced by ensuring the origin, authenticity, custody, and integrity of products. | The pair of consumers/ public and a unit of supply chain | Cognition-based Trust | - |
| Salah, Nizamuddin, Jayaraman, and Omar (2019) | The conceptual paper proposed an approach to leverage blockchain technology and smart contracts for performing soybean tracing and tracking in the agricultural supply chain. The research highlighted that adopting a blockchain-based system would eliminate the need for centralised authority and intermediaries and enable self-executing smart contracts to govern all interactions and transactions instead. Trust among relevant parties is expected to be enhanced as smart contracts provided a secured, trusted and reliable transaction and information inside the system is immutable and transparent to all stakeholders. | 1. The pair of users and blockchain-based system  2. The pair of two Supply Chain Partners - Trustless trusted scheme (TTS) | 1. Institution-based Trust  2. Cognition-based Trust | - |
| Schmidt and Wagner (2019) | The conceptual article discussed how blockchain influences supply chain relationships by shifting forms of trust from personal/ relational trust to system-based trust as the technology provides a tamper-proof of the data record. In other words, blockchain enables transactions in an environment that involved parties show no personal trust establishment, but the technology foster information sharing and transparency.  The paper also posited a proposition regarding the system-based trust enabled through blockchain which can limit opportunistic behaviour. The argument is based on the transaction cost theory. Moreover, the article stated that blockchain cannot compromise the need for relational governance which normally facilitate long-term trust and reduce opportunistic behaviour in the long run, particularly in extreme situations. | The pair of two Supply Chain Partners - Trustless trusted scheme (TTS) | 1. Institution-based Trust  2. Cognition-based Trust  3. Affect-based Trust | Long-term Oriented Trust |
| Viriyasitavat, Anuphaptrirong, and Hoonsopon (2019) | The conceptual paper provided explanations of how the breakthrough of blockchain and IoT can transform the paradigm of the current business model. Trust is one of the critical components of such transformation. Specifically, trust in the third parties to guarantee business transactions is eliminated. Yet, trust in system security, trust in data from system ledgers and trust in process executions are established through the applications of smart contracts. | 1. The pair of users and blockchain-based system  2. The pair of two Supply Chain Partners - Trustless trusted scheme (TTS) | 1. Institution-based Trust  2. Cognition-based Trust | - |
| Y. Wang, Han, and Beynon-Davies (2019) | The paper performed a systematic literature review on the applications of blockchain for supply chain management from plies of academic and practitioner literature. A handful of blockchain values are discussed as one of the main finding areas. Such values include extended visibility and traceability, enabled supply chain disintermediation, enhanced data security, and established digital trust. Blockchain helps increase the trust of transactions among supply chain participants and end-consumers through an enhancement of data security. Trust has relocated away from trusted third parties to the decentralised network.  The authors also highlighted the distinctive concept of digital trust that is built instantly and continuously adaptive when compared with traditional trust that takes years to build. This digital trust has the potential to prevent supply chain members to behave opportunistically and increase consumer confidence in the products from legitimate suppliers. | 1. The pair of users and blockchain-based system  2. The pair of two Supply Chain Partners - Inter-organisational trust reinforcement (ITR) 3. The pair of consumers/ public and a unit of supply chain | 1. Institution-based Trust  2. Cognition-based Trust | - |
| Zhao et al. (2019) | The article conducted a systematic literature analysis on blockchain applications for agri-food value chains. The findings suggested that blockchain adoption can improve agri-food value chain management in multiple aspects. Blockchain-engendered transparency which leads to an increase in trust among value chain members is one of the blockchain-driven values proposed by the authors. | The pair of two Supply Chain Partners - Inter-organisational trust reinforcement (ITR) | Cognition-based Trust | - |
| Ahmad et al. (2020) | The conceptual paper discussed the applications of blockchain for supply chain management in the aerospace and defence industry. Several key features of blockchain are highlighted including security, privacy, trust, traceability, transparency, reliability, and auditability. Concerning trust, blockchain technology is expected to establish a trusted system among untrusted entities. This is because blockchain platforms can help maintain a secure, credible, and trusted battlefield data. The application of self-executing smart contract also allows execution of transactions in a way faster and economical fashion, ensuring trust among trustless entities. Besides, the transparency of records and the traceability of components provenance also increase trust among stakeholders in the supply chain of aircraft components. | 1. The pair of users and blockchain-based system  2. The pair of two Supply Chain Partners - Trustless trusted scheme (TTS) | 1. Institution-based Trust  2. Cognition-based Trust | - |
| Alkaabi, Salah, Jayaraman, Arshad, and Omar (2020) | The paper proposed a blockchain-based solution for achieving product traceability in an additive manufacturing supply chain. The authors explained how the advantages of blockchain can be applied to help guarantee trusted and authenticated traceability of 3D printed products throughout the printing process. The blockchain-enabled trustworthy mechanisms also help overcome challenges of additive manufacturing adoption by enabling verification of parts provenance and protecting copyright and intellectual property. | 1. The pair of users and blockchain-based system  2. The pair of two Supply Chain Partners - Trustless trusted scheme (TTS) | Cognition-based Trust | - |
| Böckel, Nuzum, and Weissbrod (2020) | The paper conducted a systematic literature review of blockchain for a circular economy and addressed several topics worth investigating. Trust and verification are two major promising benefits of blockchain which could be a challenge to achieve. The authors also pointed out the remaining questions regarding trust creation between supply network participants; specifically  1) How can the trust benefit be realised when adopting blockchain?  2) How blockchain might be able to create trust if there is no trust existed in the first place? | 1. The pair of users and blockchain-based system  2. The pair of two Supply Chain Partners - Trustless trusted scheme (TTS) | Cognition-based Trust | - |
| Caldarelli, Rossignoli, and Zardini (2020) | The paper conducted a case study to investigate the impact of blockchain on organisational effectiveness and trust for sustainable supply chains. Regarding the effect of blockchain on trust, Surprisingly, the findings suggested that there is no clear evidence of how the adoption of the technology leads to trust improvement. Blockchain seems not to play the role of a trust creator, but the technology may be helpful to maintain the high level of trust which has been built over years. This means that blockchain should work only in an environment where trust is at a maximum by providing defensive mechanisms against external threats such as counterfeit products.  The paper also highlighted the controversial issue regarding blockchain and trust as early blockchain literature believed that blockchain is a means for creating trust, but more recently, scholars seem to focus on the technology capability to enable transactions performed in a trustless environment. | 1. The pair of users and blockchain-based system  2. The pair of two Supply Chain Partners - Trustless trusted scheme (TTS)/ Inter-organisational trust reinforcement (ITR) | 1. Institution-based Trust  2. Cognition-based Trust | - |
| Cha, Baek, and Kim (2020) | The paper proposed a framework of the utilisation of blockchain and key escrow encryption to optimise supply chain security for those long-lifecycle systems such as weapon systems. The paper emphasised that the proposed system can be operated under an untrusted environment and shift trust from the third party to the blockchain-based system. | 1. The pair of users and blockchain-based system  2. The pair of two Supply Chain Partners - Trustless trusted scheme (TTS) | Cognition-based Trust | Long-term Oriented Trust |
| Chang and Chen (2020) | The paper conducted a literature survey and analytical review of blockchain technology and the application of smart contracts for the supply chain management. Trust is indicated as one of the main implications of the technology implementation as blockchain features tend to help increase trust among supply chain stakeholders and reduce the dependence on traditional trusted centralised authorities and intermediaries. | 1. The pair of users and blockchain-based system  2. The pair of two Supply Chain Partners - Trustless trusted scheme (TTS) | 1. Institution-based Trust  2. Cognition-based Trust | - |
| Di Vaio and Varriale (2020) | The research performed a single case study of blockchain application in the Italian aviation industry to investigate the implication of the technology on operation management efficacy. The case finding suggested that blockchain can help promote cooperation and reduce fragmentation between industry players and the industry authority (the air traffic controllers: ATCs) by facilitating them to share important information with each other. The study also mentioned that blockchain creates trust among those relevant parties by enabling the sharing of information and data. | The pair of two Supply Chain Partners - Inter-organisational trust reinforcement (ITR) | Cognition-based Trust | - |
| Dubey, Gunasekaran, Bryde, Dwivedi, and Papadopoulos (2020) | The study empirically investigated the mechanisms of swift trust-building in the humanitarian supply chain. The researchers found a significant positive relationship between blockchain technology and swift trust as well as between operational supply chain transparency and swift trust. In sum, blockchain does enhance swift trust among participants engaged in disaster relief operations through the facilitation of verified data sharing in the network platform and an enhancement of information transparency in the supply chain. | The pair of two Supply Chain Partners - Inter-organisational trust reinforcement (ITR) | Cognition-based Trust | Swift trust |
| Garrard and Fielke (2020) | The study performed a case study of blockchain application in the prawn aquaculture industry in Australia to explore the desirable properties of a blockchain ledger in tracing authentic provenance of items which help increase the trustworthiness of the provenance and tamper risk of fraudulence. With respect to trust, the paper also highlighted that blockchain execution is free from the need to trust a third party who has the potential to be corruptible. | 1. The pair of users and blockchain-based system 2. The pair of two Supply Chain Partners - Trustless trusted scheme (TTS)3. The pair of consumers/ public and a unit of supply chain | Cognition-based Trust | - |
| H. R. Hasan, Salah, Jayaraman, Yaqoob, and Omar (2020) | The article presented the integration of blockchain with the physical internet and the benefits of such integration in which blockchain technology can help enhance transparency, trust, and security in the physical internet paradigm. The paper also emphasised the benefit of a blockchain-based physical internet system in enforcing the authentication and provenance visibility of the products in agriculture products which help enhance consumer trust. | The pair of consumers/ public and a unit of supply chain | Cognition-based Trust | - |
| Howson (2020) | The conceptual paper provided the idea of how blockchain technology can be utilised to leverage the performances of marine conservation and fishery supply chains by addressing the public distrust issue. The paper discussed the potentiality of blockchain in enhancing consumer trust and alleviating the erosion of public confidence which came from a perceived lack of transparency, frequent corruption, and abuse scandals. The authors also explained how the technology enables the automated consensus protocols which help data in the system to be verified and immutably stored. This trusted mechanism then reduce the risk of data corruption substantially. | The pair of consumers/ public and a unit of supply chain | Cognition-based Trust | - |
| Kayikci, Subramanian, Dora, and Bhatia (2020) | The research investigated the roles of blockchain in solving several problematic issues in the food supply chains. The researcher discussed the values of blockchain in fortifying technology security, supply chain transparency, and stakeholders’ trust.  With respect to trust, a blockchain consortium is expected to gather like-minded organisations together and engender a new level of trust based on a single point of truth. Blockchain also increases a level of visibility in procurement, enables trusted data storage with secured privacy, and provides more accurate data for analytics, which collectively enhance trust among all participating entities in a network. | 1. The pair of users and blockchain-based system  2. The pair of two Supply Chain Partners - Inter-organisational trust reinforcement (ITR) 3. The pair of consumers/ public and a unit of supply chain | 1. Institution-based Trust  2. Cognition-based Trust | - |
| Köhler and Pizzol (2020) | The research conducted six cases studies of blockchain use cases in the food supply chain to analyse the social and environmental implications of the technology. The authors argued that blockchain is highly likely to be coupled with other technologies in creating digitisation impacts. Specifically, blockchain direct implications on supply chain management are grounded on the enhancement of transparency, traceability, and trust. Concerning trust element, blockchain implementation helps reduce the risks of data manipulation and empower consumers to acknowledge products’ provenance, thus increasing trust among supply chain actors and end consumers. | 1. The pair of users and blockchain-based system  2. The pair of two Supply Chain Partners - Inter-organisational trust reinforcement (ITR) 3. The pair of consumers/ public and a unit of supply chain | 1. Institution-based Trust  2. Cognition-based Trust | - |
| Kopyto, Lechler, Heiko, and Hartmann (2020) | The study addressed the numerous benefits of blockchain application in the supply chain by conducting an interdisciplinary Delphi survey of the technology and supply chain experts. The authors found that experts agree unanimously on blockchain potential to reinforce trust for digital transactions. Nevertheless, the surprising key findings of the research indicated that “active trust management” between supply chain partners is still necessary even though blockchain is implemented. Counterintuitively, the trust-related advantages of blockchain cannot be directly transferred to supply chain management since the authenticity of data inputted into the system cannot be guaranteed. In other words, blockchain only enables the cryptographic proof of digital data in the system but does not deal with physical information. Therefore, trust management to reduce data manipulation before entering into the blockchain system is a prerequisite. | The pair of two Supply Chain Partners - Inter-organisational trust reinforcement (ITR) | 1. Institution-based Trust  2. Cognition-based Trust  3. Affect-based Trust | Long-term Oriented Trust |
| Kumar, Liu, and Shan (2020) | The article outlined the blockchain utilities in comparison with benefits from other inter-organisational technologies as well as explained the realistic values of blockchain by comparing its sets of advantages to the high financial cost and associated risks. The study also explained the disseminated power of blockchain infrastructure that reduces the need for trust among supply chain actors and for an  intermediary. In other words, the technology is conjectured to break the physical and temporal barriers between low-trust supply chain entities to perform transactions and trace and track products efficiently with high visibility along supply chains. Blockchain technology is then suitable for the low trust climate supply chain. While, in the situations where there is high trust among parties and low traceability and visibility needs, conventional technologies such as electronic data interchange (EDI) may be more appropriate. | The pair of two Supply Chain Partners - Trustless trusted scheme (TTS) | 1. Institution-based Trust  2. Cognition-based Trust | - |
| L'Hermitte and Nair (2020) | The paper developed a theoretical framework for the utilisation of blockchain technology for a logistics sharing system in an emergency response situation. The study also proposed the underlying process of how blockchain-based systems can help facilitate interactions between emergency responders and commercial organisations which lead to the reinforcement of trust. The authors emphasised that with the presence of blockchain, trust is moving away from the creation of a relationship-based trust system to the establishment of digital trust. Specifically, with a blockchain-based system, trust is enhanced through five differing mechanisms.  1) Blockchain platform enables information sharing to all involved parties including risk profiles of each participating entity.  2) Blockchain platform allows participants to rate their transaction partners. This rating performance then enhances trust between unknown participants.  3) Using private blockchain, only reliable and trusted parties are granted permission to access and use the system. This supports the trust enhancement as unknown parties will have no access to the emergency response systems.  4) Blockchain-enabled smart contracts can automate the resources matching and payment process as soon as pre-agreed conditions are met.  5) As blockchain exhibits pre-determined protocols that are agreed upon by all engaged parties, trust in this platform is expected to increase. | 1. The pair of users and blockchain-based system  2. The pair of two Supply Chain Partners - Inter-organisational trust reinforcement (ITR) 3. The pair of consumers/ public and a unit of supply chain | 1. Institution-based Trust  2. Cognition-based Trust  3. Affect-based Trust | Swift Trust |
| J. Li, Maiti, Springer, and Gray (2020) | The conceptual article explored the blockchain applications in the global supply chain quality management and the technology’s impacts on various associated avenues. Trust building between different parties is proposed as one of the main implications of blockchain in quality management activity. First, trust between supplier-buyers could be increased by solving the problem of information asymmetry in production processes as well as the limitations of quality inspections in the traditional supply chains. Second, end consumers’ trust in product quality could be enhanced by the visual presentation of quality test data recorded for the whole supply chain process and activities. Third, the feature of blockchain to secure data without the possibility of being tampered with could improve trust and cooperation between international companies which is crucially important in the globalisation era. | 1. The pair of two Supply Chain Partners - Inter-organisational trust reinforcement (ITR) 2. The pair of consumers/ public and a unit of supply chain | 1. Institution-based Trust  2. Cognition-based Trust | - |
| M. Li, Shao, Ye, Xu, and Huang (2020) | The article developed a framework of a blockchain-enabled logistics finance execution platform that provides a trusted runtime environment for smart contracts. The framework also paves a way for further integrated blockchain and distributed intelligent systems that enable trust among multiple stakeholders. | 1. The pair of users and blockchain-based system  2. The pair of two Supply Chain Partners - Trustless trusted scheme (TTS) | 1. Institution-based Trust  2. Cognition-based Trust | - |
| Omar, Jayaraman, Salah, Debe, and Omar (2020) | The paper proposed a blockchain-based solution for transforming vendor managed inventory (VMI) operations. The applications of smart contracts and decentralised storage systems enable bi-directional information exchange which in turn promote trust among supply chain stakeholders. In short, adopting blockchain helps streamline communication and provide a secured, transparent, trusted mode of communication among stakeholders in VMI operations. | The pair of two Supply Chain Partners - Inter-organisational trust reinforcement (ITR) | Cognition-based Trust | - |
| Ozdemir et al. (2020) | The study employed a decision-making trial and evaluation laboratory (DEMATEL) method to investigate the benefits of blockchain in the humanitarian supply chain setting. The findings revealed that blockchain adoption can help overcome the inter-organisational issues between supply chain members and donors such as lack of trust, inefficient collaboration and lack of effective donation management. | 1. The pair of two Supply Chain Partners - Inter-organisational trust reinforcement (ITR) 2. The pair of consumers/ public and a unit of supply chain | 1. Institution-based Trust  2. Cognition-based Trust | Swift trust |
| Palas and Bunduchi (2020) | The research investigated blockchain perceived values for its applications in the healthcare industry from the perspective of different stakeholders. The findings suggested that specifically for trust enhancement among supply chain partners, only experts pointed out this benefit, while vendors nor existing literature did not support the claim. Experts argued that trust improvement comes from faster data transmission and a decentralised database system which is critically important in the context where there is high corruption. | 1. The pair of users and blockchain-based system  2. The pair of two Supply Chain Partners - Inter-organisational trust reinforcement (ITR) | 1. Institution-based Trust  2. Cognition-based Trust | - |
| Pan, Pan, Song, Ai, and Ming (2020) | The research empirically analysed several issues around the relationship between blockchain technology and enterprise operational capabilities. One key finding is that blockchain does relieve the problem of information asymmetry among internal departments which contribute to the enabling of trust climate in an organisation. The technology also rearranges decision-making power through the unique consensus mechanisms which contributes to organisational synergy efficiency. Moreover, blockchain deployment helps foster trust among supply chain alliances and create a trusting supply chain ecosystem. | 1. The pair of users and blockchain-based system  2. The pair of two Supply Chain Partners - Inter-organisational trust reinforcement (ITR) | 1. Institution-based Trust  2. Cognition-based Trust | - |
| Pawar, Sonje, and Shukla (2020) | The article demonstrated a prototype of the food subsidy distribution system that is based on blockchain technology architecture. The study also expounded the operational mechanisms of such a blockchain-based system that enforces trust and transparency to the whole supply chain system. | 1. The pair of users and blockchain-based system 2. The pair of consumers/ public and a unit of supply chain | 1. Institution-based Trust  2. Cognition-based Trust | - |
| Probst (2020) | The article discussed how the adoption of blockchain, data mining, and artificial intelligence in the fisheries supply chain can alleviate the issue of trust crisis in consumers which mainly stems from the deficiency of transparency in fisheries production and trading processes. Blockchain and its smart contracts applications can be utilised to help increase transparency and availability of information across the end-to-end supply chain. This information visibility will inform consumers about the origin and production methods of their products, leading to consumers’ trust-building. | The pair of consumers/ public and a unit of supply chain | Cognition-based Trust | - |
| Pournader, Shi, Seuring, and Koh (2020) | The article conducted a systematic literature review with the co-citation analysis of blockchain applications for supply chain management and logistics. Trust is one of the key findings from the review in which it is indicated as the main value of blockchain in the supply chain context together with traceability and transparency. Trust between various transport intermediaries is expected to be increased through secured and customised information-sharing mechanisms. The paper also proposed remaining issues of trust and future research avenues which mainly associated with the buyer-supplier relationship. The authors expected that their trust will be shifted with the presence of blockchain and the trust between them might not need to be developed. Trust is also suggested as the moderator to regulate the relationship of traceability/transparency and trade. | The pair of two Supply Chain Partners - Trustless trusted scheme (TTS) | 1. Institution-based Trust  2. Cognition-based Trust | - |
| X. Qian and Papadonikolaki (2020) | The research conducted several interviews with blockchain experts to investigate how blockchain installation affects various sources and dimensions of trust in the construction supply chain. The study found that blockchain can enhance inter-organisational trust in the supply chain by facilitating supply chain participants to collaborate and establish a mechanism that helps avoid opportunistic behaviour. The findings also suggested that the form of trust is shifting from relational to system-based and cognition-based. In other words, blockchain considerably abates the need for setting up relational trust by reinforcing system-based and cognition-based trust. | The pair of two Supply Chain Partners - Inter-organisational trust reinforcement (ITR) | 1. Institution-based Trust  2. Cognition-based Trust  3. Affect-based Trust | Long-term Oriented Trust |
| J. Qian et al. (2020) | The conceptual article proposed the framework of using blockchain in the cross-border food supply chain to enhance food safety. The paper provided an example of blockchain use cases in the food trade between the European Union and China. The finding suggested that blockchain-based traceability systems can help address the transparency and security issues and foster bilateral trust in food safety during food trade. The paper also presented the details of the operation mechanism of blockchain in enhancing bilateral trust which mainly relies on the tamper-proof characteristic of data recording in the system, decentralised management, and the use of smart contracts. | 1. The pair of users and blockchain-based system  2. The pair of two Supply Chain Partners - Inter-organisational trust reinforcement (ITR) | 1. Institution-based Trust  2. Cognition-based Trust | - |
| Rogerson and Parry (2020) | The research carried out multiple case studies to unearth how blockchain enables visibility and trust in food supply chains. The authors also proposed that blockchain is likely to be executed in the supply chains of the products that consumers are willing to pay a premium price, e.g., baby food. The findings also suggested that visibility enhanced by blockchain also help reinforce trust between supply chain participants by mitigating supply chain friction and reducing the focal firm’s workload to monitor the product flows. Yet, the trust of technology, human error, and fraud are still the critical challenges for the implementation. | 1. The pair of two Supply Chain Partners - Inter-organisational trust reinforcement (ITR)2. The pair of consumers/ public and a unit of supply chain | Cognition-based Trust | - |
| Shahid et al. (2020) | The conceptual article proposed the blockchain-based agri-food supply chain which addresses several issues of the centralised traditional supply chain which include lack of transparency, accountability and auditability. The article also discussed how blockchain and smart contract helps promote trust among trading entities through the construction of a reputation system in which each entity can provide the ratings and reviews for the product owner that they purchased. This reputation system complements the trustless nature of blockchain as it helps reduce an opportunity for partners’ malicious behaviours. | 1. The pair of users and blockchain-based system  2. The pair of two Supply Chain Partners - Trustless trusted scheme (TTS) | 1. Institution-based Trust  2. Cognition-based Trust | - |
| Suhail, Hussain, Khan, and Hong (2020) | The experimental paper demonstrated the simulation of an advanced distributed ledger technological system to trace the provenance of a product and redesign the coordination among supply chain partners using trustworthy data in order to solve the counterfeit product's problem. The article also emphasised the values of data trustworthiness in solving several problems including the trust relationship between trading partners and also the waning confidence in consumer trust regarding the product journey. | 1. The pair of users and blockchain-based system  2. The pair of two Supply Chain Partners - Inter-organisational trust reinforcement (ITR) 3. The pair of consumers/ public and a unit of supply chain | 1. Institution-based Trust  2. Cognition-based Trust | Long-term Oriented Trust |
| Wan, Huang, and Holtskog (2020) | The paper conducted a systematic literature review of academic literature on blockchain for enabling information sharing in the supply chain. The findings discussed that blockchain and smart contract applications can resolve the information silo problem and fortify information sharing and data trust among members within a supply network. The study emphasised that blockchain can act as “one trusted ledger” which helps increase the information sharing between trusted and non-trusted institutions with the lower risk associated with security issues. | 1. The pair of users and blockchain-based system  2. The pair of two Supply Chain Partners - Inter-organisational trust reinforcement (ITR) | 1. Institution-based Trust  2. Cognition-based Trust | - |
| Yong et al. (2020) | The paper illustrated how a blockchain-based system coupled with machine learning technologies can establish a trusting ecosystem of the vaccine supply chain which mitigate the issues of vaccine expiration and vaccine record fraud. The authors argued that blockchain should be introduced into the vaccine supply chain to enable trust mechanism for vaccine users. The study also showed evidence of how blockchain technology and its application of smart contracts can build effective trust mechanisms between institutions and customers in managing the supply chain. | 1. The pair of two Supply Chain Partners - Inter-organisational trust reinforcement (ITR)2. The pair of consumers/ public and a unit of supply chain | 1. Institution-based Trust  2. Cognition-based Trust | - |
| Agrawal, Kumar, Pal, Wang, and Chen (2021) | The paper conceptualised and proposed the blockchain application framework for traceability in textile and clothing supply chains. The authors demonstrated how blockchain-enabled smart contracts can ensure data safety, enable traceability of exchanged data, and build a technology-based trust among the supply chain participants by using an example of an organic cotton supply chain. A technology-based trust is mainly developed through the technology features which can store and authenticate transactions without the presence of third-party authority, as well as through the blockchain-driven information sharing that enhance supply chain visibility. | 1. The pair of users and blockchain-based system  2. The pair of two Supply Chain Partners - Trustless trusted scheme (TTS) | 1. Institution-based Trust  2. Cognition-based Trust | - |
| Ahmad, Hasan, Jayaraman, Salah, and Omar (2021) | The conceptual paper proposed the blockchain applications and architecture for port logistic operations and services. The study outlined several benefits of blockchain including increasing transaction efficiency, reducing the chance of fraud, and increasing trust among stakeholders involved in the port logistics ecosystem. Concerning trust, several distinguishing characteristics of blockchain such as superior data and privacy, auditability, and transaction immutability can be applied to ports operations process which substantially strengthens trust among heterogeneous parties including exporter, port authority, customs agency, and ship carriers. | 1. The pair of users and blockchain-based system 2. The pair of two Supply Chain Partners - Inter-organisational trust reinforcement (ITR) | 1. Institution-based Trust  2. Cognition-based Trust | - |
| Ahmad, Salah, et al. (2021) | The paper demonstrated a system design along with the implementation details of a decentralised blockchain-based approach for a forward supply chain of COVID-19 medical equipment and waste management processes. The proposed blockchain-based solution offers an excellent advantage in performing the processes in a secured, transparent, traceable, and trustworthy fashion. Concerning trust, the use of self-executing smart contracts helps enforce trust among participating stakeholders as the COVID-19 medical supplies and waste management data is verified as genuine. | 1. The pair of users and blockchain-based system 2. The pair of two Supply Chain Partners - Inter-organisational trust reinforcement (ITR) | 1. Institution-based Trust  2. Cognition-based Trust | - |
| Alkhader et al. (2021) | The research proposed a blockchain-based solution that is specifically developed for manufacturing and supplying COVID-19 medical devices. Not only system architecture, but algorithms along with the platform implementation are tested. The findings demonstrated the harness of blockchain platform power in creating transparent, traceable, trustworthy supply chains of COVID-19 medical devices. | The pair of users and blockchain-based system | 1. Institution-based Trust  2. Cognition-based Trust | - |
| Alkhoori et al. (2021) | The research presented the design and implementation of a blockchain-powered smart container system that is able to monitor the conditions of vaccine shipments from departure to destination. When equipped with cloud services, this newly designed blockchain platform demonstrated its ability to detect any violations that occurred and automatically record on smart contracts which can not be tampered with. This secured and immutable storage of information can help improve trust among relevant stakeholders, especially in an inherently trustless environment. | 1. The pair of users and blockchain-based system2. The pair of two Supply Chain Partners - Trustless trusted scheme (TTS) | 1. Institution-based Trust  2. Cognition-based Trust | - |
| Asante et al. (2021) | The study comprehensively reviewed 111 published articles regarding the applications and efficacy of distributed ledger technologies (including blockchain) for the supply chain management. One of the main utilities of distributed ledger technologies is an establishment of trust among supply chain entities especially in the situation that a supply chain is full of trustless entities. Trust is enhanced through multiple mechanisms enabled by distributed ledger technologies such as the provision of data provenance, immutability features, transparent transactions, consensus protocols, and the application of smart contracts. All of these mechanisms can collectively promote trust among trustless entities. | 1. The pair of users and blockchain-based system2. The pair of two Supply Chain Partners - Trustless trusted scheme (TTS) | 1. Institution-based Trust  2. Cognition-based Trust | - |
| Baharmand, Maghsoudi, and Coppi (2021) | The study carried out several interviews with humanitarian practitioners and academicians to consolidate various issues of blockchain for the humanitarian supply chain. Concerning trust, the paper evidently found that blockchain has the potential to strengthen both commitment trust (trust that is based on contractual agreements) and swift trust, even though interviewees referred to commitment trust more than swift trust. Specifically, blockchain can enhance commitment trust through the facilitation of smart contracts’ features. Besides, blockchain also fortifies swift trust through the enhancement of supply chain transparency. | The pair of two Supply Chain Partners - Inter-organisational trust reinforcement (ITR) | 1. Institution-based Trust  2. Cognition-based Trust | Swift trust |
| Bai et al. (2021) | The article proposed a model of blockchain-based green data supply chain in the agricultural industry based on a game-theoretic approach. The blockchain-based model is grounded on the trust management mechanisms in which the trust values of each sensor can be stored in the blockchain system. Basically, the behaviours of the entities in the system will affect its trust value. This means that those entities with malicious behaviours will be punished and reduced trust score. | 1. The pair of users and blockchain-based system2. The pair of two Supply Chain Partners - Trustless trusted scheme (TTS) | 1. Institution-based Trust  2. Cognition-based Trust | - |
| Bamakan, Faregh, and ZareRavasan (2021) | The paper proposed a newly designed supply chain performance evaluation system based on blockchain technology and adaptive network-based fuzzy inference systems (ANFIS) techniques, called Di-ANFIS architecture. This system architecture integrates blockchain with the application of IoT technology and smart contracts. The developed system platform is believed to be intelligent, tamper-proof, trustable which can remedy the issues of trust and security in the supply chain. Also, the proposed system is decentralised which eliminates a need for trusted intermediaries. | 1. The pair of users and blockchain-based system2. The pair of two Supply Chain Partners - Trustless trusted scheme (TTS) | 1. Institution-based Trust  2. Cognition-based Trust | - |
| S. Cao et al. (2021) | The article adopted a design science approach to demonstrate how consumer trust is strengthened in the cross-border beef supply chain between Australia and China by implementing blockchain-based solutions. The authors proposed the integration of self-governance mechanisms combined with regulatory authorities is conjectured to fortify trust and confidence in the food supply chain. These mechanisms include the novel human-machine reconcile mechanism which facilitates the responsibility sharing between agriculture, supply chain members, and external stakeholders to trace and track credential data along the supply chain and share them to end consumers. The product traceability information can be delivered with blockchain credentialed videos to enhance consumers’ trust and confidence. | The pair of consumers/ public and a unit of supply chain | 1. Institution-based Trust  2. Cognition-based Trust | - |
| Casino et al. (2021) | The study developed a blockchain-based, distributed trustless and secure architecture for food supply chain traceability and assessed the feasibility of the approach using a case study from a dairy firm. Various advantages of blockchain are discussed including improved efficiency, quality, resilience, and trust. Specifically, for the trust concept, the three certain characteristics of blockchain namely auditability, accountability, and immutability lead to an enhancement of trust in the traceability system. | 1. The pair of users and blockchain-based system2. The pair of two Supply Chain Partners - Trustless trusted scheme (TTS) | 1. Institution-based Trust  2. Cognition-based Trust | - |
| Centobelli, Cerchione, Del Vecchio, Oropallo, and Secundo (2021) | The study investigated the blockchain implementation for circular supply chains and designed the blockchain platform using an integrated Triple Retry framework to address three main effects of the blockchain (trust, traceability, and transparency) on the three reverse supply chain processes (recycle, redistribute, remanufacture). Concerning trust, the implementation of blockchain could modify the concept of trust among supply chain parties by replacing trust in a centralised authority or individual partners with trust in blockchain technology. In other words, trust in individual partners is integrated by the trust in the blockchain. The trust in blockchain technology mainly comes from the mechanism of decentralised and distributed ledger, an increase in information disclosure, guaranteed responsibility attribution, and compromised vulnerability of information flows and transactions. The authors also emphasised that the blockchain platform has a positive effect on both the trustor and the trustee in the circular economy. | The pair of two Supply Chain Partners - Inter-organisational trust reinforcement (ITR) | 1. Institution-based Trust  2. Cognition-based Trust  3. Affect-based Trust | - |
| Cocco et al. (2021) | The article presented the design and implementation of a blockchain-based system for supply chain management in a particular Italian bread manufacturing process. The blockchain-embedded system is integrated with the application of IoT and radio-frequency identification (RFID) technologies. The authors proposed that this newly designed system creates a trustless environment, in which trust is shifted from placing in public or private firms to cryptography, mathematical operations and on the network. | 1. The pair of users and blockchain-based system  2. The pair of two Supply Chain Partners - Trustless trusted scheme (TTS) | 1. Institution-based Trust  2. Cognition-based Trust | - |
| de Boissieu, Kondrateva, Baudier, and Ammi (2021) | The research conducted multiple case studies to explore the utilities of blockchain implementation for supply chains in the luxury industry. The findings highlighted several merits of blockchain merits such as brand reputation protection and customers’ trust establishment. Specifically, promoting the accessibility of products’ data, such as products’ origin by having a certificate attached to the product on the blockchain platform, is likely to increase end-users’ trust especially in the second-hand luxury market. | The pair of consumers/ public and a unit of supply chain | 1. Cognition-based Trust  2. Affect-based Trust | - |
| Garaus and Treiblmaier (2021) | The study empirically proposed and validated the mediating role of trust in the relationship between blockchain-based traceability systems and the consumers’ choice of retailors. The findings revealed that a blockchain-equipped traceability system does significantly impacts consumers’ trust, and subsequently influences their retailer choice. The finding also confirmed that consumers exhibit higher levels of trust in retailers who execute blockchain-based systems compared to those using a traditional traceability system. | The pair of consumers/ public and a unit of supply chain | 1. Cognition-based Trust  2. Affect-based Trust | - |
| Hijazi, Perera, Calheiros, and Alashwal (2021) | The article conducted a systematic literature review and presented a rationale for applying blockchain with building information modelling (BIM) for enabling a reliable digital deliverable in construction supply chains. As trust remains the most crucial challenge in the construction supply chain, blockchain could come to play role in resolving this issue. One of the blockchain key strengths is that the technology enables the trusted exchange of data directly without an intermediary. This strength could be very useful in low-trust environments such as in the construction supply chain. When equipped with BIM that acts as the source of data, the authors project that this approach could pave the way for a single source of truth system. | 1. The pair of users and blockchain-based system 2. The pair of two Supply Chain Partners - Trustless trusted scheme (TTS) | 1. Institution-based Trust  2. Cognition-based Trust | - |
| Hu, Huang, Huang, and Su (2021) | The paper illustrated the framework of the combination of blockchain and edge computing in leveraging consumer trust in the organic agricultural supply chains. Blockchain is believed to be a solution for a severe trust crisis of consumers. The technology can help build trust in both perspectives of system trust (trust in labelling and certification processes, normally rooted in institutions) and technology trust (the technological system’s performance and quality attributes). The blockchain-based system provides a trusted data flow, creating a more transparent, authentic and trustworthy supply chain management. Furthermore, a blockchain-enabled consensus mechanism can automatically reconcile between trading parties even though they seem not to trust each other, creating a trustless traceability system. | 1. The pair of users and blockchain-based system2. The pair of two Supply Chain Partners - Trustless trusted scheme (TTS)3. The pair of consumers/ public and a unit of supply chain | 1. Institution-based Trust  2. Cognition-based Trust | - |
| Hunt, Narayanan, and Zhuang (2021) | The article performed a systematic literature review on blockchain applications in humanitarian supply chains and proposed several avenues for future research. The study highlighted several blockchain features which offer a great deal of promise to humanitarian supply chain management including auditability, immutability, and trust. Several ways in which blockchain implementation could foster trust between participants within relief networks have been discussed including promoting transparency and accountability in information sharing as well as executing smart contracts with a peer rating system to facilitate coordination between involved parties. Future research should pay attention to an issue of the entity that takes ownership of the blockchain system and responsibility for potential hazards. This ownership issue could directly affect trust within the network. | The pair of two Supply Chain Partners - Inter-organisational trust reinforcement (ITR) | Cognition-based Trust | Swift trust |
| Joo and Han (2021) | The study developed the structural equation model and empirically tested the relationship between blockchain-enabled trust, its three determinants, and user satisfaction within food supply chains. The authors found that distributed trust driven from blockchain plays a role as a mediator between the three influencing determinants (transparency, traceability, and security) and the satisfaction among supply chain participants. Therefore, a blockchain-based system helps enhance trust between supply chain actors including producers, processors, distributors, retailers, and consumers.  The paper also applied grounded theory to discuss trust-free systems based on blockchain technology and the commonalities and differences of system-like trust and human-like trust in the blockchain. The findings suggested that distributed trust along supply chains developed through the implementation of blockchain technology resembles the system-like trust (consists of reliability, functionality, and helpfulness) more than the human-like trust (consists of integrity, ability, and benevolence). | The pair of two Supply Chain Partners - Inter-organisational trust reinforcement (ITR) | 1. Cognition-based Trust  2. Affect-based Trust | - |
| Karamchandani, Srivastava, Kumar, and Srivastava (2021) | The study empirically conducted a survey to examine blockchain perceived roles in the manufacturing industry. Consumer trust is flagged up as one of the main values generated by blockchain adoption as blockchain enable nearly real-time, trustworthy information about purchased products. The article also stated that when the technology reaches its maturity, consumers may consider purchasing products only from those trustworthy organisations using blockchain. Moreover, the authors posited that blockchain would considerably improve trust among participants engaged in cross-border trade activities through the provision of trustworthy real-time data. | 1. The pair of two Supply Chain Partners - Inter-organisational trust reinforcement (ITR)2. The pair of consumers/ public and a unit of supply chain | 1. Institution-based Trust  2. Cognition-based Trust | - |
| Khan, Imtiaz, Parvaiz, Hussain, and Bae (2021) | The research empirically examined the implications of blockchain and IoT in humanitarian supply chains. The findings suggested that the integration of blockchain and IoT helps enhance public trust and coordination in the supply chain during disaster management crises. The result also indicated that transparency mediates the relationship between technology execution and supply chain performance (trust and coordination). In other words, blockchain increases transparency which subsequently enhances public trust. | 1. The pair of two Supply Chain Partners - Inter-organisational trust reinforcement (ITR)2. The pair of consumers/ public and a unit of supply chain | Cognition-based Trust | Swift trust |
| Lee and Yeon (2021) | The authors conducted a pilot experiment to test how blockchain-based e-commerce platforms could help increase trust among retail platforms, manufacturing companies, and end customers. The findings contributed to the blockchain potential to resolve the issue of counterfeit products and lack of traceability and openness of information in e-commerce transactions which could harm platform stakeholders’ trustworthiness. | 1. The pair of two Supply Chain Partners - Inter-organisational trust reinforcement (ITR)2. The pair of consumers/ public and a unit of supply chain | Cognition-based Trust | - |
| K. Li, Lee, and Gharehgozli (2021) | The paper reviewed and synthesised research on blockchain applications in food supply chains. Several blockchain benefits and distinct characteristics which could collectively contribute to an enhancement of trust between supply chain members are highlighted. These include the shared immutable data in the system, unprecedented visibility across the end-to-end chain, traceability of financial resources and goods, as well as reduction in food fraud and waste. | The pair of two Supply Chain Partners - Inter-organisational trust reinforcement (ITR) | Cognition-based Trust | - |
| Z. Li et al. (2021) | The article conducted a literature review of blockchain articles published recently between 2017 and 2020 and analysed multi-perspective of blockchain research. The implication of blockchain in building trust is also pointed out. Specifically, the article briefly explained the system trust construction process through the blockchain-enabled consensus mechanism and cryptography algorithms. The article also flagged up the importance of the need for legal supervision and regulation of blockchain which will promote the trust of consumers and investors in the long run. | 1. The pair of users and blockchain-based system 2. The pair of two Supply Chain Partners - Trustless trusted scheme (TTS) 3. The pair of consumers/ public and a unit of supply chain | 1. Institution-based Trust  2. Cognition-based Trust | Long-term Oriented Trust |
| L. Liu, Li, and Jiang (2021) | The paper analysed how the utilisation of blockchain platform finance could help solve compromised trust that manufacturers and commercial banks placed on deep-tier retailers. This lack of trust issue usually happens to those retailers that are small and medium enterprises (SMEs) with limited financial capital and need to seek loans from core enterprises and financial institutions. With an increase in information visibility enabled by blockchain, SMEs’ credibility and trustworthiness are enhanced, and they are more likely to obtain finance from large enterprises and financial institutions. | The pair of two Supply Chain Partners - Inter-organisational trust reinforcement (ITR) | 1. Institution-based Trust  2. Cognition-based Trust | - |
| W. Liu, Shao, Wu, and Qiao (2021) | The paper conducted a systematic literature review on information and communications technologies (ICTs) and blockchain applications for developing precision agriculture. The authors highlighted that blockchain benefits that are outstanding from other ICTs are the reinforcement of transparency and smart contract executions which leads to enhanced trust among supply chain actors as well as increased operational efficiency. | The pair of two Supply Chain Partners - Inter-organisational trust reinforcement (ITR) | 1. Institution-based Trust  2. Cognition-based Trust | - |
| Lu et al. (2021) | The research proposed and validated the system architecture of a blockchain-enabled supply chain management in the construction industry. The result of four case studies suggested that the blockchain-embedded system allows accurate data to be retrieved against malicious data and the corresponding reputation scores are computed. This reputation evaluation mechanism helps screen out malicious construction data effectively, which in turn increase the trustworthiness of the system. One of the main contributions in this study is to show the power of blockchain applications as trustworthy hardware oracles for construction supply chain management. | 1. The pair of users and blockchain-based system 2. The pair of two Supply Chain Partners - Trustless trusted scheme (TTS) | Institution-based Trust | - |
| Luzzani, Grandis, Frey, and Capri (2021) | The study explored the adoption of blockchain for addressing sustainability issues in wine supply chains. The results suggested that blockchain enables information collection which could help monitor and improve sustainability such as information related to soil and water features, climate condition, treatment with pesticides and fertilizers, production process, traceability, labour and human rights. The improvement in data transparency consequently leads to an enhancement of end-consumers’ trust placed on producers which is reflected in sales growth. | The pair of consumers/ public and a unit of supply chain | Cognition-based Trust | - |
| Musamih et al. (2021) | The article presented the blockchain system architecture and detailed algorithms for leveraging smart contracts and enabling efficient product traceability in healthcare supply chains. The designed blockchain-based system exhibits the superior security system for data stored inside which make it impossible to be manipulated in every stage of product transfer across supply chains, leading to the establishment of a trustless system of transparency.  The study also demonstrated how the trustworthy track and trace system enabled by blockchain can help guarantee data provenance and reduce the chance of counterfeit drugs occurrence, which consequently increase consumer trust in supply chains. | 1. The pair of users and blockchain-based system 2. The pair of two Supply Chain Partners - Trustless trusted scheme (TTS) 3. The pair of consumers/ public and a unit of supply chain | 1. Institution-based Trust  2. Cognition-based Trust | - |
| Patro, Ahmad, Yaqoob, Salah, and Jayaraman (2021) | The article presented a blockchain-based approach for product recall management in the automotive supply chain. The authors also discussed the implementation details and the potentials of this system to overcome current problematic issues regarding transparency, traceability, reliability, audit, security, and trust of the product recall process. The authors highlighted that blockchain helps ensure that users perform their activities in the system in a trustworthy manner. | The pair of users and blockchain-based system | Institution-based Trust | - |
| Pawar, Sonje, and Shukla (2021) | The article discussed how strategic objectives of a food subsidy distribution system could be achieved through the implementation of designed architecture and operational mechanisms based on blockchain technology. The findings suggested that with the robust application of blockchain and the use of smart contracts, the relationships between multiple stakeholders could be governed through smart contracts, and trust and transparency could be enforced in the food subsidy system. Holistically, blockchain is capable of developing a food subsidy distribution system with greater trust, accountability, and transparency. | 1. The pair of users and blockchain-based system2. The pair of two Supply Chain Partners - Trustless trusted scheme (TTS) | Institution-based Trust | - |
| Pranto, Noman, Mahmud, and Haque (2021) | The paper demonstrated a system that integrated blockchain with IoT for enabling smart agriculture. The authors clearly explained how the applications of smart contracts help regulate interaction among contributing parties along the supply chain. The advantage of using the blockchain-based system is discussed. Trust establishment among parties is mentioned as one of the main advantages which can be achieved through smart contracts execution. Specifically, the self-executed contract builds in the trustless rigid contract between players involved and automates the tracing process, so avoiding manipulation of data. | 1. The pair of users and blockchain-based system2. The pair of two Supply Chain Partners - Trustless trusted scheme (TTS) | Institution-based Trust | - |
| Rana et al. (2021) | The study developed blockchain-based solutions for supply chain management to improve performance in various aspects. Blockchain shows potential to overcome several long-standing issues in supply chain management as the technology is capable of providing secured traceability and immutable information which establishes trust to relevant stakeholders.  The authors also comprehensively compared the blockchain-based system with the traditional supply chain system. While the trust-related issues seem to present frequently with a centralised approach, a blockchain-based system could bring about the collaborative environment in which trust is increased. | The pair of two Supply Chain Partners - Inter-organisational trust reinforcement (ITR) | Cognition-based Trust | - |
| Rejeb, Keogh, Simske, Stafford, and Treiblmaier (2021) | The paper conducted a narrative literature review on the great promise of blockchain potentials for supply chain collaboration. Through several mechanisms, blockchain adoption can positively reinforce collaboration among different alliances including strengthening communicative relationships, facilitating auditability, improving accountability, and enhancing trust in business-to-business relationships. From the perspective of trust, the electronic and immutable characteristics of blockchain networks lead to the built-in trust of the system. Also, since the blockchain-based system is decentralised, the need for assessing the trustworthiness of intermediaries and other entities is eliminated. Supply chain participants then perform data exchange through the mechanism of trusted networked collaboration facilitated by blockchain. In sum, blockchain helps enhance trust through information sharing, substitute trust relationships needed in the traditional supply chain with the trust in the technology, and address trust issues between partners with incentive alignment by executing smart contracts. | 1. The pair of users and blockchain-based system 2. The pair of two Supply Chain Partners - Trustless trusted scheme (TTS) | 1. Institution-based Trust  2. Cognition-based Trust | - |
| Runzel, Hassler, Rogers, Formato, and Cazier (2021) | The paper proposed smart honey supply chain systems based on blockchain technology which can fortify food security and food safety as well as reduce honey fraud. The study illustrated that the proposed blockchain-based system can strengthen consumer trust in agricultural products, especially for quality and adulteration, through the execution of a trustworthy traceability system and the establishment of a chain of custody. | The pair of consumers/ public and a unit of supply chain | Cognition-based Trust | - |
| Subramanian and Thampy (2021) | The research mainly discussed the applications of blockchain for a pre-owned, online electric vehicle market. As in the present time, there is asymmetric information of electric conveyance history, battery charging capabilities, charging historical records, and other information that could affect decision making. The adoption of the Ethereum blockchain could address this issue by assigning stakeholders including electric vehicle manufacturers, charging stations, battery manufacturers, road conveyers ascendancy to act as a distributed ledger and track the vehicle records. Consequently, the employment of blockchain predicated solutions could increase transparency in a futuristic supply chain and engender trust in electric vehicle buyers. | 1. The pair of users and blockchain-based system2. The pair of two Supply Chain Partners - Trustless trusted scheme (TTS)3. The pair of consumers/ public and a unit of supply chain | 1. Institution-based Trust  2. Cognition-based Trust | - |
| Surjandari, Yusuf, Laoh, and Maulida (2021) | The study illustrated the application of distributed ledger, blockchain in halal food supply chains. The improvement in transparency, information disclosure between supply chain alliances as well as trust among supply chain participants in the food items are expected to be increased from the secured, immutable attribute of blockchain. In addition, the article also emphasised the necessity for halal certification agencies to adopt blockchain in order to increase the trust and authenticity of the halal brand. | 1. The pair of users and blockchain-based system  2. The pair of two Supply Chain Partners - Inter-organisational trust reinforcement (ITR) 3. The pair of consumers/ public and a unit of supply chain | 1. Institution-based Trust  2. Cognition-based Trust | - |
| Tezel, Febrero, Papadonikolaki, and Yitmen (2021) | The article investigated the utilisation of blockchain in construction supply chains in three different projects: supply chain payments, reverse auction-based tendering for procurement, and asset tokenisation for project financing. A broad set of blockchain-enabled benefits are identified including streamlined, transparent transactions and rational trust-building. The results suggest that one of the blockchain common benefits in the three models is an enhancement of trust placed on the system as well as the trust placed between the project stakeholders. The authors also emphasised that with the presence of blockchain, supply chain entities can trust information, yet not necessarily each other. | 1. The pair of users and blockchain-based system 2. The pair of two Supply Chain Partners - Trustless trusted scheme (TTS) | 1. Institution-based Trust  2. Cognition-based Trust  3. Affect-based Trust | - |
| Treiblmaier and Sillaber (2021) | The paper discussed various topics revolving around the applications of blockchain on e-commerce and also identified 19 high-level research questions to inspire future research on this topic. An increase in trust is one of the impacts engendered by using blockchain technology. Trust discussed in the paper comes in various perspectives including trustless trusted exchange relations among supply chain alliances which can operate without dedicated intermediaries and trust enhancement in system’s users due to the reinforcement of system security. | 1. The pair of users and blockchain-based system 2. The pair of two Supply Chain Partners - Trustless trusted scheme (TTS) | 1. Institution-based Trust  2. Cognition-based Trust | - |
| Tseng and Shang (2021) | The study discussed how blockchain technology can be adopted as an intermediary using an explanation of the trust mechanism. Such a trust mechanism includes the automatic transmission of data from point to point which help minimise human error or tampering, and the change in audit activities which can only be performed under safe conditions. | 1. The pair of users and blockchain-based system 2. The pair of two Supply Chain Partners - Trustless trusted scheme (TTS) | 1. Institution-based Trust  2. Cognition-based Trust | - |
| Uddin (2021) | The conceptual paper discussed the use of a blockchain-based traceability system in the drug supply chains to counter counterfeit drugs. The research explained the mechanisms in which blockchain can create a trusted environment between untrusted parties by providing the identity management service and tracking the transactions and their workflows amongst relevant stakeholders in the supply chain. With this traceability system, the need for a trusted centralised authority is then eliminated. The study also emphasised that, through the capability to store immutable records, the provision of consensus mechanisms, the use of private keys, and the execution of decentralised networks to enable transparent communication, blockchain can help strengthen trust between untrusted parties in the pharmaceutical supply chain. | 1. The pair of users and blockchain-based system2. The pair of two Supply Chain Partners - Trustless trusted scheme (TTS) | 1. Institution-based Trust  2. Cognition-based Trust | - |
| Vashistha et al. (2021) | The article developed a prototype of a blockchain-centric solution for verifying authentic electronic devices from those counterfeit products which often come from untrusted entities in supply chains, such as outsourced foundries and system integrators. With the capability of blockchain technology, all supply chain participants can access device provenance records and apply a consensus mechanism to each certain electronic device. This increases the trustworthiness of the system and compromises the chance of counterfeit product experiences. | 1. The pair of users and blockchain-based system2. The pair of two Supply Chain Partners - Trustless trusted scheme (TTS) | Institution-based Trust | - |
| Velmovitsky, Bublitz, Fadrique, and Morita (2021) | The article conducted a literature review to discuss the multitudes of blockchain solutions used in the healthcare industry including health insurance, genomics, and drug and food supply chains. For supply chain management, using blockchain can help participants obtains several benefits such as an increase in transparency, elimination of counterfeit products, and minimisation of trust concerns among parties. Regarding trust establishment, there are several mechanisms discussed including the process of consent management and the use of smart contracts. | 1. The pair of users and blockchain-based system  2. The pair of two Supply Chain Partners - Inter-organisational trust reinforcement (ITR) | 1. Institution-based Trust  2. Cognition-based Trust | - |
| H. Wang, Zhang, Ying, and Zhao (2021) | The study empirically examines the implications of a blockchain tracing system on consumer behaviours. The findings revealed that blockchain adoption does show a significant, positive impact on supply chain transparency and process management, which subsequently contribute to the enhancement of consumer service and trust. Concerning trust, multiple factors enabled by blockchain adoption collectively leads to trust reinforcement including product quality, safety and authenticity as well as firms’ brand image. | The pair of consumers/ public and a unit of supply chain | Cognition-based Trust | - |
| Z. Wang, Zheng, Jiang, and Tang (2021) | The article designed a stylised, blockchain-based supply chain that enables data-sharing between trading entities. The proposed system overcomes the issue of privacy concerns, data misuse, asymmetric data possession, and untrustworthy data-sharing mechanisms. In short, the designed architecture of blockchain enables trustworthy information sharing in the supply chain. | The pair of users and blockchain-based system | 1. Institution-based Trust  2. Cognition-based Trust | - |
| Xu and Yang (2021) | The analytical paper analysed blockchain applications for logistics port transportation by utilising a logical data optimisation model. The paper also applied the inductive method for reasoning and discussed the potential utilities of blockchain in this logistics and transport context. One of the blockchain benefits raised in this paper is the establishment of trust among providers as the product quality is truthfully checked and the origin of the products is ensured during transportation. | 1. The pair of users and blockchain-based system2. The pair of two Supply Chain Partners - Inter-organisational trust reinforcement (ITR) | Cognition-based Trust | - |
| Yang, Zhang, and Shi (2021) | The research developed a game-theoretic model to examine player decisions when blockchain is implemented and proposed optimal solutions regarding the blockchain adoption strategies for players involved in the food retailing platform. This blockchain-based platform could foster consumers’ trust through information visibility and address the issue of serious health and safety concerns in food supply chains during the COVID-19 outbreak. | The pair of consumers/ public and a unit of supply chain | Cognition-based Trust | - |
| X. Yang et al. (2021) | The paper illustrated the design of a blockchain-equipped traceability system for storage and query of product information in food and vegetable supply chains. An increase in consumers’ trust is one of the products obtained from using this blockchain-based traceability system. As detailed information about agricultural products is displayed to consumers, their concerns regarding food safety are reduced and their trust placed on companies is increased. Also, the variability and reliability of product quality ensured by the real-time tracking capabilities of the system help resolve the trust problem of participants in fruit supply chains. | 1. The pair of two Supply Chain Partners - Inter-organisational trust reinforcement (ITR) 2. The pair of consumers/ public and a unit of supply chain | Cognition-based Trust | - |
| Zhu, Peko, Sundaram, and Piramuthu (2021) | The article investigated how the convergence of IoT and blockchain technology can establish new agile supply chain frameworks. Specifically, the capability of IoT in providing fine-granular real-time data from related sensor networks and the capability of blockchain in enabling visibility and traceability collectively support agile operation. With the presence of these two advanced technologies, necessary low-level information used to establish an agile supply chain can be ensured in a trustworthy manner. | The pair of users and blockchain-based system | Cognition-based Trust | - |

**References**

Agrawal, T. K., Kumar, V., Pal, R., Wang, L., & Chen, Y. (2021). Blockchain-based framework for supply chain traceability: A case example of textile and clothing industry. *Computers & Industrial Engineering, 154*, 107130. doi:10.1016/j.cie.2021.107130

Ahmad, R. W., Hasan, H., Jayaraman, R., Salah, K., & Omar, M. (2021). Blockchain applications and architectures for port operations and logistics management. *Research in Transportation Business & Management*, 100620. doi:10.1016/j.cie.2020.106982

Ahmad, R. W., Hasan, H., Yaqoob, I., Salah, K., Jayaraman, R., & Omar, M. (2020). Blockchain for aerospace and defense: Opportunities and open research challenges. *Computers & Industrial Engineering*, 106982. doi:10.1016/j.cie.2020.106982

Ahmad, R. W., Salah, K., Jayaraman, R., Yaqoob, I., Omar, M., & Ellahham, S. (2021). Blockchain-Based Forward Supply Chain and Waste Management for COVID-19 Medical Equipment and Supplies. *IEEE Access, 9*, 44905-44927. doi:10.1109/ACCESS.2021.3066503

Alkaabi, N., Salah, K., Jayaraman, R., Arshad, J., & Omar, M. (2020). Blockchain-based traceability and management for additive manufacturing. *IEEE Access, 8*, 188363-188377. doi:10.1109/ACCESS.2020.3031536

Alkhader, W., Salah, K., Sleptchenko, A., Jayaraman, R., Yaqoob, I., & Omar, M. (2021). Blockchain-Based Decentralized Digital Manufacturing and Supply for COVID-19 Medical Devices and Supplies. *IEEE Access, 9*, 137923-137940. doi:10.1109/ACCESS.2021.3118085

Alkhoori, O., Hassan, A., Almansoori, O., Debe, M., Salah, K., Jayaraman, R., . . . Rehman, M. H. U. (2021). Design and Implementation of CryptoCargo: A Blockchain-Powered Smart Shipping Container for Vaccine Distribution. *IEEE Access, 9*, 53786-53803. doi:10.1109/ACCESS.2021.3070911

Asante, M., Epiphaniou, G., Maple, C., Al-Khateeb, H., Bottarelli, M., & Ghafoor, K. Z. (2021). Distributed Ledger Technologies in Supply Chain Security Management: A Comprehensive Survey. *IEEE Transactions on Engineering Management*. doi:10.1109/TEM.2021.3053655

Baharmand, H., Maghsoudi, A., & Coppi, G. (2021). Exploring the application of blockchain to humanitarian supply chains: insights from Humanitarian Supply Blockchain pilot project. *International Journal of Operations & Production Management*. doi:10.1108/IJOPM-12-2020-0884

Bai, Y., Fan, K., Zhang, K., Cheng, X., Li, H., & Yang, Y. (2021). Blockchain-based trust management for agricultural green supply: A game theoretic approach. *Journal of Cleaner Production, 310*, 127407. doi:10.1016/j.jclepro.2021.127407

Bamakan, S. M. H., Faregh, N., & ZareRavasan, A. (2021). Di-ANFIS: an integrated blockchain–IoT–big data-enabled framework for evaluating service supply chain performance. *Journal of Computational Design and Engineering, 8*(2), 676-690. doi:10.1093/jcde/qwab007

Benčić, F. M., Skočir, P., & Žarko, I. P. (2019). DL-Tags: DLT and smart tags for decentralized, privacy-preserving, and verifiable supply chain management. *IEEE Access, 7*, 46198-46209. doi:10.1109/ACCESS.2019.2909170

Böckel, A., Nuzum, A.-K., & Weissbrod, I. (2020). Blockchain for the Circular Economy: Analysis of the Research-Practice Gap. *Sustainable Production and Consumption*. doi:10.1016/j.spc.2020.12.006

Caldarelli, G., Rossignoli, C., & Zardini, A. (2020). Overcoming the blockchain oracle problem in the traceability of non-fungible products. *Sustainability, 12*(6), 2391. doi:10.3390/su12062391

Cao, B., Li, Y., Zhang, L., Zhang, L., Mumtaz, S., Zhou, Z., & Peng, M. (2019). When Internet of Things meets blockchain: Challenges in distributed consensus. *IEEE Network, 33*(6), 133-139. doi:10.1109/MNET.2019.1900002

Cao, S., Powell, W., Foth, M., Natanelov, V., Miller, T., & Dulleck, U. (2021). Strengthening consumer trust in beef supply chain traceability with a blockchain-based human-machine reconcile mechanism. *Computers and Electronics in Agriculture, 180*, 105886. doi:10.1016/j.compag.2020.105886

Casino, F., Kanakaris, V., Dasaklis, T. K., Moschuris, S., Stachtiaris, S., Pagoni, M., & Rachaniotis, N. P. (2021). Blockchain-based food supply chain traceability: a case study in the dairy sector. *International Journal of Production Research, 59*(19), 5758-5770. doi:10.1080/00207543.2020.1789238

Centobelli, P., Cerchione, R., Del Vecchio, P., Oropallo, E., & Secundo, G. (2021). Blockchain technology for bridging trust, traceability and transparency in circular supply chain. *Information & Management*, 103508. doi:10.1016/j.im.2021.103508

Cha, S., Baek, S., & Kim, S. (2020). Blockchain Based Sensitive Data Management by Using Key Escrow Encryption System From the Perspective of Supply Chain. *IEEE Access, 8*, 154269-154280. doi:10.1109/ACCESS.2020.3017871

Chang, S. E., & Chen, Y. (2020). When blockchain meets supply chain: A systematic literature review on current development and potential applications. *IEEE Access, 8*, 62478-62494. doi:10.1109/ACCESS.2020.2983601

Cocco, L., Mannaro, K., Tonelli, R., Mariani, L., Lodi, M. B., Melis, A., . . . Fanti, A. (2021). A Blockchain-Based Traceability System in Agri-Food SME: Case Study of a Traditional Bakery. *IEEE Access, 9*, 62899-62915. doi:10.1109/ACCESS.2021.3074874

de Boissieu, E., Kondrateva, G., Baudier, P., & Ammi, C. (2021). The use of blockchain in the luxury industry: supply chains and the traceability of goods. *Journal of Enterprise Information Management*. doi:10.1108/JEIM-11-2020-0471

Di Vaio, A., & Varriale, L. (2020). Blockchain technology in supply chain management for sustainable performance: Evidence from the airport industry. *International Journal of Information Management, 52*, 102014. doi:10.1016/j.ijinfomgt.2019.09.010

Dubey, R., Gunasekaran, A., Bryde, D. J., Dwivedi, Y. K., & Papadopoulos, T. (2020). Blockchain technology for enhancing swift-trust, collaboration and resilience within a humanitarian supply chain setting. *International Journal of Production Research, 58*(11), 3381-3398. doi:10.1080/00207543.2020.1722860

Garaus, M., & Treiblmaier, H. (2021). The influence of blockchain-based food traceability on retailer choice: The mediating role of trust. *Food Control, 129*, 108082. doi:10.1016/j.foodcont.2021.108082

Garrard, R., & Fielke, S. (2020). Blockchain for trustworthy provenances: A case study in the Australian aquaculture industry. *Technology in Society, 62*, 101298. doi:10.1016/j.techsoc.2020.101298

Hasan, H., AlHadhrami, E., AlDhaheri, A., Salah, K., & Jayaraman, R. (2019). Smart contract-based approach for efficient shipment management. *Computers & Industrial Engineering, 136*, 149-159. doi:10.1016/j.cie.2019.07.022

Hasan, H. R., Salah, K., Jayaraman, R., Yaqoob, I., & Omar, M. (2020). Blockchain Architectures for Physical Internet: A Vision, Features, Requirements, and Applications. *IEEE Network*. doi:10.1109/MNET.021.2000442

Hijazi, A. A., Perera, S., Calheiros, R. N., & Alashwal, A. (2021). Rationale for the integration of BIM and blockchain for the construction supply chain data delivery: A systematic literature review and validation through focus group. *Journal of Construction Engineering and Management, 147*(10), 03121005. doi:10.1061/(ASCE)CO.1943-7862.0002142

Howson, P. (2020). Building trust and equity in marine conservation and fisheries supply chain management with blockchain. *Marine Policy, 115*, 103873. doi:10.1016/j.marpol.2020.103873

Hu, S., Huang, S., Huang, J., & Su, J. (2021). Blockchain and edge computing technology enabling organic agricultural supply chain: A framework solution to trust crisis. *Computers & Industrial Engineering, 153*, 107079. doi:10.1016/j.cie.2020.107079

Hunt, K., Narayanan, A., & Zhuang, J. (2021). Blockchain in humanitarian operations management: A review of research and practice. *Socio-Economic Planning Sciences*, 101175. doi:10.1016/j.seps.2021.101175

Joo, J., & Han, Y. (2021). An Evidence of Distributed Trust in Blockchain-Based Sustainable Food Supply Chain. *Sustainability, 13*(19), 10980. doi:10.3390/su131910980

Juma, H., Shaalan, K., & Kamel, I. (2019). A survey on using blockchain in trade supply chain solutions. *IEEE Access, 7*, 184115-184132. doi:10.1109/ACCESS.2019.2960542

Karamchandani, A., Srivastava, S. K., Kumar, S., & Srivastava, A. (2021). Analysing perceived role of blockchain technology in SCM context for the manufacturing industry. *International Journal of Production Research*, 1-32. doi:10.1080/00207543.2021.1883761

Kayikci, Y., Subramanian, N., Dora, M., & Bhatia, M. S. (2020). Food supply chain in the era of Industry 4.0: blockchain technology implementation opportunities and impediments from the perspective of people, process, performance, and technology. *Production Planning & Control*, 1-21. doi:10.1080/09537287.2020.1810757

Khan, M., Imtiaz, S., Parvaiz, G. S., Hussain, A., & Bae, J. (2021). Integration of Internet-of-Things with Blockchain Technology to Enhance Humanitarian Logistics Performance. *IEEE Access*. doi:10.1109/ACCESS.2021.3054771

Köhler, S., & Pizzol, M. (2020). Technology assessment of blockchain-based technologies in the food supply chain. *Journal of Cleaner Production, 269*, 122193. doi:10.1016/j.jclepro.2020.122193

Kopyto, M., Lechler, S., Heiko, A., & Hartmann, E. (2020). Potentials of blockchain technology in supply chain management: Long-term judgments of an international expert panel. *Technological Forecasting and Social Change, 161*, 120330. doi:10.1016/j.techfore.2020.120330

Kumar, A., Liu, R., & Shan, Z. (2020). Is blockchain a silver bullet for supply chain management? Technical challenges and research opportunities. *Decision Sciences, 51*(1), 8-37. doi:10.1111/deci.12396

L'Hermitte, C., & Nair, N. K. C. (2020). A blockchain‐enabled framework for sharing logistics resources during emergency operations. *Disasters*. doi:10.1111/disa.12436

Lee, H., & Yeon, C. (2021). Blockchain-Based Traceability for Anti-Counterfeit in Cross-Border E-Commerce Transactions. *Sustainability, 13*(19), 11057. doi:doi.org/10.3390/su131911057

Li, J., Maiti, A., Springer, M., & Gray, T. (2020). Blockchain for supply chain quality management: challenges and opportunities in context of open manufacturing and industrial internet of things. *International Journal of Computer Integrated Manufacturing, 33*(12), 1321-1355. doi:10.1080/0951192X.2020.1815853

Li, K., Lee, J.-Y., & Gharehgozli, A. (2021). Blockchain in food supply chains: a literature review and synthesis analysis of platforms, benefits and challenges. *International Journal of Production Research*, 1-20. doi:10.1080/00207543.2021.1970849

Li, M., Shao, S., Ye, Q., Xu, G., & Huang, G. Q. (2020). Blockchain-enabled logistics finance execution platform for capital-constrained E-commerce retail. *Robotics and Computer-Integrated Manufacturing, 65*, 101962. doi:10.1016/j.rcim.2020.101962

Li, Z., Zhong, R. Y., Tian, Z., Dai, H.-N., Barenji, A. V., & Huang, G. Q. (2021). Industrial Blockchain: A state-of-the-art Survey. *Robotics and Computer-Integrated Manufacturing, 70*, 102124. doi:10.1016/j.rcim.2021.102124

Liu, L., Li, Y., & Jiang, T. (2021). Optimal strategies for financing a three-level supply chain through blockchain platform finance. *International Journal of Production Research*, 1-18. doi:10.1080/00207543.2021.2001601

Liu, W., Shao, X.-F., Wu, C.-H., & Qiao, P. (2021). A systematic literature review on applications of information and communication technologies and blockchain technologies for precision agriculture development. *Journal of Cleaner Production*, 126763. doi:10.1016/j.jclepro.2021.126763

Longo, F., Nicoletti, L., Padovano, A., d'Atri, G., & Forte, M. (2019). Blockchain-enabled supply chain: An experimental study. *Computers & Industrial Engineering, 136*, 57-69. doi:10.1016/j.cie.2019.07.026

Lu, W., Li, X., Xue, F., Zhao, R., Wu, L., & Yeh, A. G. (2021). Exploring smart construction objects as blockchain oracles in construction supply chain management. *Automation in Construction, 129*, 103816. doi:10.1016/j.autcon.2021.103816

Luzzani, G., Grandis, E., Frey, M., & Capri, E. (2021). Blockchain Technology in Wine Chain for Collecting and Addressing Sustainable Performance: An Exploratory Study. *Sustainability, 13*(22), 12898. doi:10.3390/su132212898

Meng, W., Tischhauser, E. W., Wang, Q., Wang, Y., & Han, J. (2018). When intrusion detection meets blockchain technology: a review. *IEEE Access, 6*, 10179-10188. doi:10.1109/ACCESS.2018.2799854

Montecchi, M., Plangger, K., & Etter, M. (2019). It’s real, trust me! Establishing supply chain provenance using blockchain. *Business Horizons, 62*(3), 283-293. doi:10.1016/j.bushor.2019.01.008

Musamih, A., Salah, K., Jayaraman, R., Arshad, J., Debe, M., Al-Hammadi, Y., & Ellahham, S. (2021). A Blockchain-Based Approach for Drug Traceability in Healthcare Supply Chain. *IEEE Access, 9*, 9728-9743. doi:10.1109/ACCESS.2021.3049920

Omar, I. A., Jayaraman, R., Salah, K., Debe, M., & Omar, M. (2020). Enhancing vendor managed inventory supply chain operations using blockchain smart contracts. *IEEE Access, 8*, 182704-182719. doi:10.1109/ACCESS.2020.3028031

Ozdemir, A. I., Erol, I., Ar, I. M., Peker, I., Asgary, A., Medeni, T. D., & Medeni, I. T. (2020). The role of blockchain in reducing the impact of barriers to humanitarian supply chain management. *The International Journal of Logistics Management*. doi:10.1108/IJLM-01-2020-0058

Palas, M. J. U., & Bunduchi, R. (2020). Exploring interpretations of blockchain's value in healthcare: a multi-stakeholder approach. *Information Technology & People*. doi:10.1108/ITP-01-2019-0008

Pan, X., Pan, X., Song, M., Ai, B., & Ming, Y. (2020). Blockchain technology and enterprise operational capabilities: An empirical test. *International Journal of Information Management, 52*, 101946. doi:10.1016/j.ijinfomgt.2019.05.002

Pärssinen, M., Kotila, M., Rumin, R. C., Phansalkar, A., & Manner, J. (2018). Is blockchain ready to revolutionize online advertising? *IEEE Access, 6*, 54884-54899. doi:10.1109/ACCESS.2018.2872694

Patro, P. K., Ahmad, R. W., Yaqoob, I., Salah, K., & Jayaraman, R. (2021). Blockchain-based solution for product recall management in the automotive supply chain. *IEEE Access*. doi:10.1109/ACCESS.2021.3137307

Pawar, R. S., Sonje, S. A., & Shukla, S. (2020). Food subsidy distribution system through Blockchain technology: a value focused thinking approach for prototype development. *Information Technology for Development*, 1-29. doi:10.1080/02681102.2020.1841714

Pawar, R. S., Sonje, S. A., & Shukla, S. (2021). Food subsidy distribution system through Blockchain technology: a value focused thinking approach for prototype development. *Information Technology for Development, 27*(3), 470-498. doi:10.1080/02681102.2020.1841714

Pournader, M., Shi, Y., Seuring, S., & Koh, S. L. (2020). Blockchain applications in supply chains, transport and logistics: a systematic review of the literature. *International Journal of Production Research, 58*(7), 2063-2081. doi:10.1080/00207543.2019.1650976

Pranto, T. H., Noman, A. A., Mahmud, A., & Haque, A. B. (2021). Blockchain and smart contract for IoT enabled smart agriculture. *PeerJ Computer Science, 7*, e407. doi:10.7717/peerj-cs.407

Probst, W. N. (2020). How emerging data technologies can increase trust and transparency in fisheries. *ICES Journal of Marine Science, 77*(4), 1286-1294. doi:10.1093/icesjms/fsz036

Qian, J., Wu, W., Yu, Q., Ruiz‐Garcia, L., Xiang, Y., Jiang, L., . . . Yang, P. (2020). Filling the trust gap of food safety in food trade between the EU and China: An interconnected conceptual traceability framework based on blockchain. *Food and Energy Security, 9*(4), e249. doi:10.1002/fes3.249

Qian, X., & Papadonikolaki, E. (2020). Shifting trust in construction supply chains through blockchain technology. *Engineering, Construction and Architectural Management*. doi:10.1108/ECAM-12-2019-0676

Rana, S. K., Kim, H.-C., Pani, S. K., Rana, S. K., Joo, M.-I., Rana, A. K., & Aich, S. (2021). Blockchain-Based Model to Improve the Performance of the Next-Generation Digital Supply Chain. *Sustainability, 13*(18), 10008. doi:10.3390/su131810008

Rejeb, A., Keogh, J. G., Simske, S. J., Stafford, T., & Treiblmaier, H. (2021). Potentials of blockchain technologies for supply chain collaboration: a conceptual framework. *The International Journal of Logistics Management*. doi:10.1108/IJLM-02-2020-0098

Rogerson, M., & Parry, G. C. (2020). Blockchain: case studies in food supply chain visibility. *Supply Chain Management: An International Journal*. doi:10.1108/SCM-08-2019-0300

Runzel, M. A., Hassler, E. E., Rogers, R., Formato, G., & Cazier, J. A. (2021). Designing a Smart Honey Supply Chain for Sustainable Development. *IEEE Consumer Electronics Magazine*. doi:10.1109/MCE.2021.3059955

Salah, K., Nizamuddin, N., Jayaraman, R., & Omar, M. (2019). Blockchain-based soybean traceability in agricultural supply chain. *IEEE Access, 7*, 73295-73305. doi:10.1109/ACCESS.2019.2918000

Schmidt, C. G., & Wagner, S. M. (2019). Blockchain and supply chain relations: A transaction cost theory perspective. *Journal of Purchasing and Supply Management, 25*(4), 100552. doi:10.1016/j.pursup.2019.100552

Shahid, A., Almogren, A., Javaid, N., Al-Zahrani, F. A., Zuair, M., & Alam, M. (2020). Blockchain-based agri-food supply chain: A complete solution. *IEEE Access, 8*, 69230-69243. doi:10.1109/ACCESS.2020.2986257

Subramanian, G., & Thampy, A. S. (2021). Implementation of Hybrid Blockchain in a Pre-owned Electric Vehicle Supply Chain. *IEEE Access*. doi:10.1109/ACCESS.2021.3084942

Suhail, S., Hussain, R., Khan, A., & Hong, C. S. (2020). Orchestrating product provenance story: When IOTA ecosystem meets electronics supply chain space. *Computers in Industry, 123*, 103334. doi:10.1016/j.compind.2020.103334

Surjandari, I., Yusuf, H., Laoh, E., & Maulida, R. (2021). Designing a Permissioned Blockchain Network for the Halal Industry using Hyperledger Fabric with multiple channels and the raft consensus mechanism. *Journal of Big Data, 8*(1), 1-16. doi:10.1186/s40537-020-00405-7

Tezel, A., Febrero, P., Papadonikolaki, E., & Yitmen, I. (2021). Insights into Blockchain Implementation in Construction: Models for Supply Chain Management. *Journal of Management in Engineering, 37*(4), 04021038. doi:10.1061/(ASCE)ME.1943-5479.0000939

Treiblmaier, H., & Sillaber, C. (2021). The impact of blockchain on e-commerce: a framework for salient research topics. *Electronic Commerce Research and Applications, 48*, 101054. doi:10.1016/j.elerap.2021.101054

Tseng, C.-T., & Shang, S. S. (2021). Exploring the Sustainability of the Intermediary Role in Blockchain. *Sustainability, 13*(4), 1936. doi:10.3390/su13041936

Uddin, M. (2021). Blockchain Medledger: A Hyperledger Fabric Enabled Drug Traceability System for Counterfeit Drugs in Pharmaceutical Industry. *International Journal of Pharmaceutics*, 120235. doi:10.1016/j.ijpharm.2021.120235

Vashistha, N., Hossain, M. M., Shahriar, M. R., Farahmandi, F., Rahman, F., & Tehranipoor, M. (2021). eChain: A Blockchain-enabled Ecosystem for Electronic Device Authenticity Verification. *IEEE Transactions on Consumer Electronics*. doi:10.1109/TCE.2021.3139090

Velmovitsky, P. E., Bublitz, F. M., Fadrique, L. X., & Morita, P. P. (2021). Blockchain Applications in Health Care and Public Health: Increased Transparency. *JMIR Medical Informatics, 9*(6), e20713. doi:10.2196/20713

Viriyasitavat, W., Anuphaptrirong, T., & Hoonsopon, D. (2019). When blockchain meets Internet of Things: Characteristics, challenges, and business opportunities. *Journal of industrial information integration, 15*, 21-28. doi:10.1016/j.jii.2019.05.002

Wan, P. K., Huang, L., & Holtskog, H. (2020). Blockchain-enabled information sharing within a supply chain: A systematic literature review. *IEEE Access, 8*, 49645-49656. doi:10.1109/ACCESS.2020.2980142

Wang, H., Zhang, M., Ying, H., & Zhao, X. (2021). The impact of blockchain technology on consumer behavior: a multimethod study. *Journal of Management Analytics, 8*(3), 371-390. doi:10.1080/23270012.2021.1958264

Wang, Y., Han, J. H., & Beynon-Davies, P. (2019). Understanding blockchain technology for future supply chains: a systematic literature review and research agenda. *Supply Chain Management: An International Journal*.

Wang, Z., Zheng, Z., Jiang, W., & Tang, S. (2021). Blockchain‐Enabled Data Sharing in Supply Chains: Model, Operationalization and Tutorial. *Production and Operations Management*. doi:10.1111/poms.13356

Xu, X., & Yang, Y. (2021). Analysis of logistics port transportation efficiency evaluation based on the block chain technology. *Annals of Operations Research*, 1-18. doi:10.1007/s10479-021-04379-4

Yang, L., Zhang, J., & Shi, X. (2021). Can blockchain help food supply chains with platform operations during the COVID-19 outbreak? *Electronic Commerce Research and Applications, 49*, 101093. doi:10.1016/j.elerap.2021.101093

Yang, X., Li, M., Yu, H., Wang, M., Xu, D., & Sun, C. (2021). A Trusted Blockchain-Based Traceability System for Fruit and Vegetable Agricultural Products. *IEEE Access, 9*, 36282-36293. doi:10.1109/ACCESS.2021.3062845

Yong, B., Shen, J., Liu, X., Li, F., Chen, H., & Zhou, Q. (2020). An intelligent blockchain-based system for safe vaccine supply and supervision. *International Journal of Information Management, 52*, 102024. doi:10.1016/j.ijinfomgt.2019.10.009

Zhao, G., Liu, S., Lopez, C., Lu, H., Elgueta, S., Chen, H., & Boshkoska, B. M. (2019). Blockchain technology in agri-food value chain management: A synthesis of applications, challenges and future research directions. *Computers in Industry, 109*, 83-99. doi:10.1016/j.compind.2019.04.002

Zhu, X. N., Peko, G., Sundaram, D., & Piramuthu, S. (2021). Blockchain-Based Agile Supply Chain Framework with IoT. *Information Systems Frontiers*, 1-16. doi:10.1007/s10796-021-10114-y
